# Institutional digitalization and digital stress: a four-wave observational study of teacher-reported AI-collaborative teaching in physical education

**DOI:** 10.3389/fpsyg.2026.1973587

**Published:** 2026-10-15

**Authors:** Xiangyu Wang, Xiaoyun Wang

**Affiliations:** 1 School of Physical Education, Sichuan Normal University, Chengdu, China; 2 Key Laboratory of Sports and Health Tourism Technology Research and Development, Department of Culture and Tourism of Sichuan Province, Chengdu, China; 3 School of Physical Education, Southwest Petroleum University, Chengdu, China

**Keywords:** cross-level interaction, digital self-efficacy, digital stress, human-artificial intelligence collaboration, institutional digitalization, job demands-resources theory, physical education teachers, technostress

## Abstract

**Introduction:**

How institutional digitalization relates to teachers’ experience of work and subsequent artificial intelligence (AI)-supported practice remains unclear.

**Methods:**

Drawing on job demands-resources principles, we examined these relationships in a four-wave observational cohort of 961 physical education teachers from 120 Chinese institutions. We linked an organizational-source digitalization proxy to teacher questionnaires and a study-specific teacher-reported score of AI involvement in teaching tasks. Analyses adjusted for baseline practice and separated between-institution from teacher-relative associations.

**Results:**

Two of five primary tests met the Holm-adjusted criterion. The stress-related product of between-institution coefficients was negative (−0.099; pointwise 95% confidence interval [−0.184, −0.033]). The positive digitalization–stress association was weaker among teachers with higher digital self-efficacy relative to colleagues (interaction coefficient −0.101; [−0.168, −0.037]) but remained positive at the low, average, and high self-efficacy levels examined. The adjusted digitalization–practice association, appraisal-related coefficient product, and appraisal interaction did not meet the corrected criterion.

**Discussion:**

The findings connect the organization and experience of work with reported AI practice, emphasizing perceived digital work pressure and relative self-efficacy while complementing accounts centered on technology acceptance.

## 1 Introduction

AI-supported teaching involves both technology use and the organization of teachers’ work. Digital systems become part of decisions about how tasks are performed, which information deserves attention, and how colleagues coordinate their activities. Work-design research places these arrangements at the center of employees’ experience of technology: digital systems can accompany greater autonomy and useful feedback, as well as additional monitoring, learning requirements, and interruptions (Wang et al., 2020; Parker and Grote, 2022). Understanding AI-supported teaching therefore requires attention to the working conditions in which teachers use these systems.

Research on teachers’ use of AI links trust, perceived usefulness, competence, and professional learning to acceptance and reported use (Choi et al., 2023; Dogan et al., 2025; Hazzan-Bishara et al., 2025; Viberg et al., 2025). Reviews describe a range of instructional applications and practical constraints on their implementation (Crompton et al., 2024; Ifenthaler et al., 2024; Martin et al., 2024). Occupational research broadens this perspective by examining AI collaboration in relation to engagement, proactive behavior, and career sustainability (Kong et al., 2023; Ding et al., 2025; Sun et al., 2025; Sun et al., 2026). Work on teachers’ use of digital data likewise attends to individual and school contexts (Michos et al., 2025). Teacher technostress studies, in turn, associate digitally related pressure with intentions and well-being (Joo et al., 2016; Pagán-Garbín et al., 2024; Truța et al., 2023). These literatures suggest that favorable evaluations of technology can coexist with difficult experiences of digital work. Explaining this coexistence calls for an account that connects teachers’ evaluations and perceived pressure to the organizational context in which AI enters their work.

Job demands-resources (JD-R) theory provides a framework for examining these experiences. It distinguishes work characteristics that require sustained effort from those that support goal attainment, help manage demands, or promote development (Demerouti et al., 2001; Bakker and Demerouti, 2007, 2017). Digital job demands are digital technology-related work requirements that call for sustained effort, such as digital reporting obligations, learning complex systems, and managing interruptions. Practical support and discretion over tasks may serve as resources. Employees’ evaluations and psychological responses reflect their experience of these working conditions. Technostress research makes a related distinction among technological stressors, users’ appraisals and responses, and organizational arrangements that support users (Ragu-Nathan et al., 2008; Tarafdar et al., 2019). Together, these perspectives guide our examination of institutional digitalization, teachers’ evaluations of AI, and perceived digital work pressure.

In our JD-R-informed application, the reported digitalization summary (RDS) is an organizational-source proxy for the broader institutional context, rather than a direct measure of particular job demands or resources. Functional appraisal captures teachers’ evaluations of AI’s adaptability, quality, and personal utility. Digital stress captures perceived digital work pressure, with adapted items covering felt strain, appraised workload, effort, and disruption. These scores describe different aspects of teachers’ experience of digital work. Digital self-efficacy (DSE), defined as confidence in performing digital teaching tasks, is a personal resource that may help explain differences among colleagues. The later AI-collaborative teaching score (AI-CTB) captures reported task practice. This conceptual map connects institutional context with teachers’ experience and reported practice.

The theoretical issue is how a digitalized organizational context can offer possibilities for AI-supported teaching while also being associated with demanding work experiences. Functional appraisal and digital stress are not opposite ends of a single continuum: recognizing AI’s value does not establish that teachers find digital work manageable, and experiencing pressure does not imply that they see no value in AI. JD-R principles make this distinction important because access to technology does not by itself establish its value as a resource for accomplishing a particular task. Examining appraisal and stress together addresses how perceived functional value and perceived work pressure relate to subsequent practice. It connects the opportunities associated with digitalization to the experience of doing the work.

Physical education (PE) makes the fit between digital information and work organization especially salient. Teachers attend to moving students, coordinate equipment, and make instructional judgments within limited lesson time. Digital feedback may assist movement diagnosis, while device preparation and output checking compete for attention during the same tasks. PE research documents varied instructional uses of technology alongside practical barriers to integration (Jastrow et al., 2022; Wallace et al., 2023; Østerlie et al., 2026; Pürgstaller et al., 2025; Saiz-González et al., 2025a, 2025b). The study items give these issues concrete referents, including device calibration, motor-performance assessment, and classroom-risk recognition. PE thus offers an informative setting for examining how digital tools fit embodied, time-constrained work and how teachers experience the accompanying pressure.

AI-CTB situates AI use in tasks that call on teachers’ professional judgment. Adapted from employee-AI collaboration research (Kong et al., 2023), its five items assess reported AI involvement in decision making, prediction, diagnosis, information evaluation, and recognition of risks or improvement opportunities. In PE, these activities include interpreting movement information and considering instructional responses. This study-specific teacher-reported practice score therefore complements research on acceptance and adoption intentions by focusing on AI involvement in the work teachers perform.

A digitalized work context may support AI involvement in established teaching tasks by providing access to relevant information and coordinated systems (Wang et al., 2020; Parker and Grote, 2022). Such arrangements can make AI more accessible within existing work routines, creating opportunities to use its outputs in professional judgments. As a broad proxy for that context, RDS provides a basis for expecting teachers in more digitalized institutions to report greater subsequent AI involvement in their work. H1 concerns this prospective association after accounting for baseline practice and measured background differences.

*H1*. Higher T1 RDS is positively associated with the T3 AI-CTB score after adjustment for the source-matched baseline score, DSE, and measured teacher- and institution-level covariates.

Functional appraisal concerns whether teachers see AI as useful for the work they undertake. Evaluations of adaptability, information quality, and personal utility inform judgments about AI’s place in work (Venkatesh et al., 2003; Park et al., 2024). A more digitalized institutional context may provide opportunities to encounter useful applications and assess their relevance to teaching tasks. This reasoning suggests higher average functional appraisal among teachers in institutions with higher RDS. More favorable institution-average appraisal may also be associated with greater subsequent AI involvement in reported teaching tasks. H2 links these expectations at the institutional level while distinguishing recognition of functional value from its practical realization in teaching.

*H2*. Institutions with higher T1 RDS will show higher average T2 functional appraisal, and higher institution-average functional appraisal will be associated with higher T3 AI-CTB scores.

Digital stress concerns the pressure teachers experience in digitalized work, including the effort involved in meeting digital requirements. Technostress research identifies workload, connectivity expectations, and complexity as possible sources of difficult work experiences (Ragu-Nathan et al., 2008; Tarafdar et al., 2019). Studies of teachers, including those working in university PE, associate digital teaching requirements with reported stress and burnout (Joo et al., 2016; Truța et al., 2023; Yang et al., 2026). Institutions with higher RDS may therefore show higher average perceived digital work pressure alongside the opportunities considered in H2. Greater pressure may coincide with less discretionary attention for evaluating outputs or incorporating AI into time-sensitive lessons, suggesting a negative association with later AI-CTB. H3 connects the expected positive RDS–stress association with the expected negative stress–practice association.

*H3*. Institutions with higher T1 RDS will show higher average T2 digital stress, and higher institution-average digital stress will be associated with lower T3 AI-CTB scores.

DSE may help explain why teachers working in the same institution differ in these experiences. It captures confidence in carrying out digital tasks (Compeau and Higgins, 1995; Hatlevik and Hatlevik, 2018; Tondeur et al., 2023). Teachers with higher DSE relative to their colleagues may more readily envisage how AI can be applied to their tasks and recognize useful applications in a digitalized context. This supports the stronger positive RDS–appraisal association proposed in H4a. Confidence in handling unfamiliar systems and troubleshooting under time constraints may also make digital requirements feel more manageable, supporting the weaker positive RDS–stress association proposed in H4b. DSE thus has two distinct proposed functions: recognizing functional opportunities and managing perceived work pressure. Both hypotheses concern a teacher’s confidence relative to colleagues who share the institutional setting.

*H4a*. The positive association between T1 RDS and T2 functional appraisal is stronger among teachers whose DSE is higher relative to colleagues within the same institution.

*H4b*. The positive association between T1 RDS and T2 digital stress is weaker among teachers whose DSE is higher relative to colleagues within the same institution.

Together, these hypotheses examine the relationship between institutional digitalization and reported AI-supported practice while distinguishing the functional value teachers attribute to AI from the pressure they experience in digital work. DSE adds a personal-resource perspective on why these experiences may differ among colleagues. By considering organizational context, both forms of experience, and relative confidence, the study applies JD-R principles to a work behavior often examined primarily in terms of technology acceptance. PE provides a setting in which the practical relevance of digital information depends on its fit with embodied, time-constrained professional activity. Figure 1 summarizes the measurement sequence and analytic levels.

**Figure 1:**
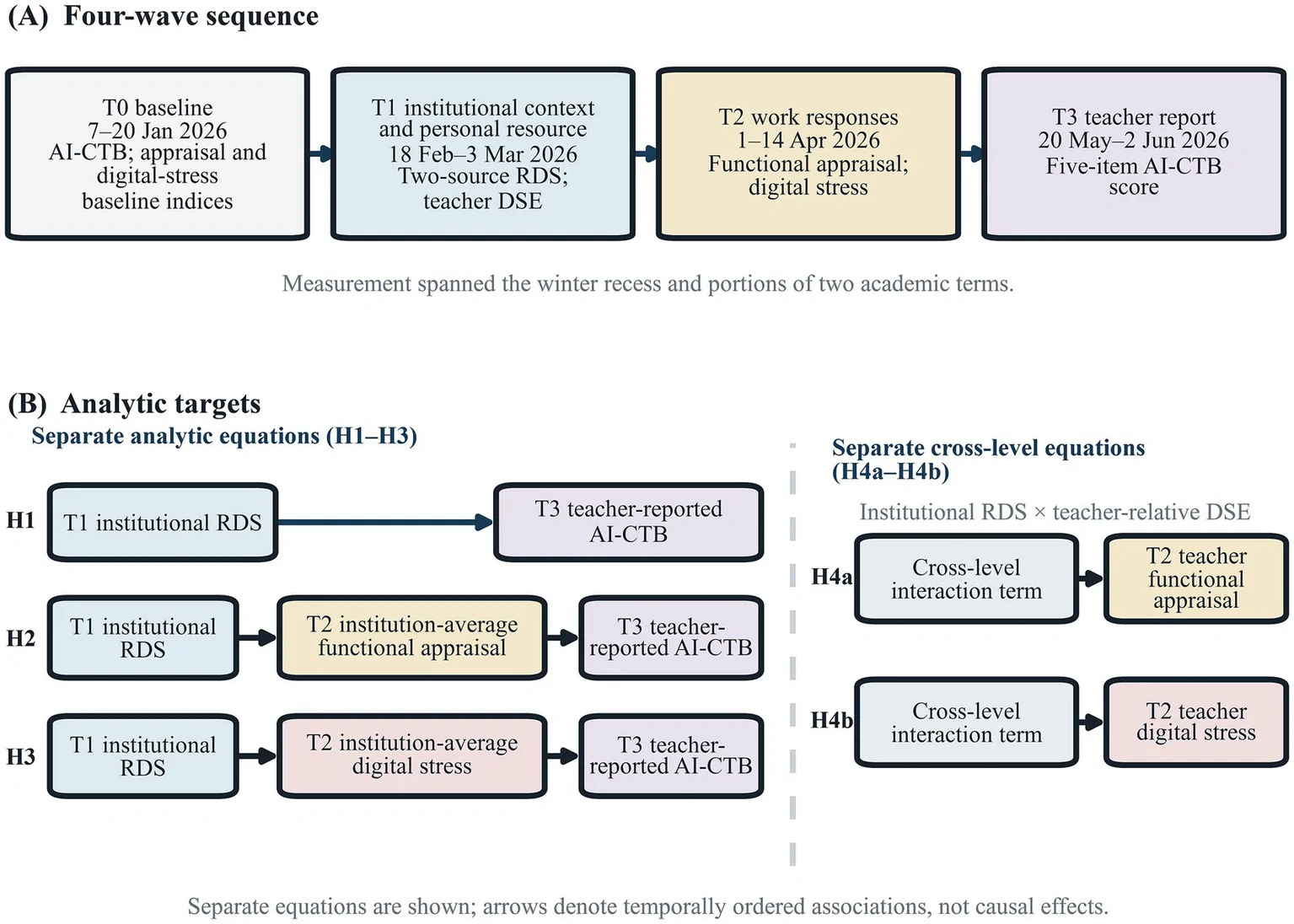
Measurement schedule and analytic framework. **(A)** Planned measurement windows; actual completion timestamps for the organizational-source RDS components were unavailable. **(B)** H1 is the RDS coefficient in a baseline-adjusted observed-score OLS equation. H2 and H3 are products of between-institution coefficients from separate equations. H4a and H4b are RDS × teacher-relative DSE interactions in separate response equations. Arrows show the proposed order of associations. Baseline scores, lower-order terms, the nonfocal institution-mean DSE interaction, and covariates are omitted for clarity. RDS, organizational-source reported digitalization proxy; DSE, digital self-efficacy; AI-CTB, study-specific teacher-reported AI involvement in teaching tasks.

## 2 Materials and methods

### 2.1 Study design and participants

This prospective four-wave observational study involved adult PE teachers. The Ethics Review Committee of the School of Physical Education, Sichuan Normal University, reviewed the protocol and granted exemption from full ethics review on 5 January 2026 (reference 2026LSTY010), before recruitment began on 7 January 2026. The reviewed protocol specified written participant information and informed consent indicated by voluntary questionnaire submission, without collecting a separate signed consent form. Responses were linked using study codes. Optional follow-up contact details were stored separately, restricted to the research team, and excluded from the analysis files. Results are reported in aggregate.

We combined de-identified teacher-questionnaire scores with two archived organizational-source summaries. Their source labels, institution head and information technology lead, follow the archived headers.

The source-tracking file contained 1,474 teacher records from 126 institutions. The complete-record analytic cohort comprised 961 teachers in 120 institutions (65.2% of source records). The other 513 records fell into mutually exclusive archived categories: no T1 response (77), no T2 response (96), no T3 response (84), invalid matching code (54), historical quality control (59), and upstream eligibility or record completeness (143). These categories describe record status rather than a sequential attrition process. Decision rules were unavailable for the last two categories, which together contained 202 records. Table 1 summarizes the archived classifications.

**Table 1:** Record classifications in the source-tracking file.

Record status	Source-tracking category	n
Not retained	No T1 response	77
Not retained	Undocumented historical quality-control label	59
Not retained	No T2 response	96
Not retained	No T3 response	84
Not retained	Invalid matching code	54
Not retained	Upstream non-questionnaire eligibility or record-completeness exclusion	143
Retained	Complete questionnaire analysis record	961
Institution coverage	126 source institutions; 120 with retained teacher records	120

Teacher counts sum to 1,474. The categories are mutually exclusive archived classifications. Of the 513 excluded records, 311 had missing-wave or invalid-linkage labels. Decision rules could not be reconstructed for 59 historical quality-control labels and 143 upstream eligibility or record-completeness labels (202 records). The source file contained 126 institutions, of which 120 had retained teacher records. Reasons for the six institutions without retained teachers were unavailable. Wave-specific response rates could not be calculated from these classifications.

Age differed between the analysis and tracking files for 174 retained teachers, and the archive did not establish which version was correct. The primary models retained analysis-file age for traceability. Omitting age changed no focal point estimate by more than 0.000097 (Supplementary Section S1.15 and Supplementary Tables S20 and S28).

The archive lacked a sampling frame, invitation denominators, a complete wave-by-wave response history, and detailed eligibility decisions for all records. Wave-specific response rates, selection probabilities, and the reasons for the loss of six institutions were therefore unavailable. Excluded records also lacked the focal scores and most covariates needed for imputation or selection adjustment. We analyzed the complete records to define a reproducible cohort; the available information did not establish random missingness. Table 2 describes this cohort, to which the findings directly apply.

**Table 2:** Characteristics of the analytic cohort (*N* = 961).

Characteristic	Category/statistic	n or mean (SD)	% or range
Age, analysis-file version	Mean (SD)	39.46 (7.84)	24–58
Age, source-tracking version	Mean (SD)	38.78 (7.80)	24–58
Teaching experience, years	Mean (SD)	13.92 (8.12)	1–35
Weekly physical education class hours	Mean (SD)	13.17 (3.64)	4–22
Average class size	Mean (SD)	38.88 (8.35)	15–65
Prior technology experience, 1–7	Mean (SD)	4.23 (0.90)	1.00–6.96
Gender	Male	507	52.8
Gender	Female	454	47.2
Region	Eastern	302	31.4
Region	Central	249	25.9
Region	Western	330	34.3
Region	Northeast	80	8.3
Institutional stage	Primary school	291	30.3
Institutional stage	Junior high school	255	26.5
Institutional stage	High school	163	17.0
Institutional stage	Higher education	252	26.2
Institutional sector	Public	830	86.4
Institutional sector	Private	131	13.6
Institutional location	Central urban	391	40.7
Institutional location	General urban	280	29.1
Institutional location	County town	194	20.2
Institutional location	Township	96	10.0
Highest educational qualification	Junior college or below	22	2.3
Highest educational qualification	Bachelor’s	583	60.7
Highest educational qualification	Master’s	302	31.4
Highest educational qualification	Doctoral	54	5.6
Prior AI-related training	Yes	351	36.5
Prior AI-related training	No	610	63.5

Percentages may not sum to 100 because of rounding. Broad region and institutional-stage categories preserve de-identification. Age differed between the archived versions for 174 records; primary models retained analysis-file age for traceability. Omitting age changed no focal point estimate by more than 0.000097.

### 2.2 Data-collection sequence

Prospective teacher recruitment and enrollment occurred during the T0 baseline survey from 7 to 20 January 2026. The T1 design window for the organizational-source summaries, teacher-perceived digitalization conditions, and DSE ran from 18 February to 3 March 2026; the T2 appraisal and digital-stress wave ran from 1 to 14 April 2026; and the T3 teacher-reported AI-CTB wave ran from 20 May to 2 June 2026. The T0, T1, and T2 start dates were 6 weeks apart; the T2-to-T3 start-date interval was 7 weeks.

The January–June measurement period included the winter recess and portions of two academic terms.

### 2.3 Measures

#### 2.3.1 Organizational-source digitalization proxy

RDS was assigned to T1 and constructed from two archived organizational-source summary fields, each ranging from 1 to 7, for 126 institutions. The Chinese headers described the fields as school-head-report and information-technology-lead-report SDO means; we use institution-head and IT-lead as descriptive source labels. Each field was standardized across all 126 institutions using the population standard deviation. The two standardized values were averaged with equal weights, and the composite was restandardized. Institution identifiers linked these source-frame scores to teachers in the 120 retained institutions. The archive lacked component items, original scoring documentation, respondent identifiers, verification of respondent roles, and records establishing independent completion or source-specific completion times. RDS is therefore interpreted as an organizational-source proxy for reported institutional digitalization.

The two source summaries correlated at *r* = 0.819 across all 126 institutions and *r* = 0.804 across the 120 retained institutions. RDS also correlated at *r* = 0.881 with the institution mean of a separate 16-item teacher-perceived digitalization block. This block drew on the digital-orientation domains in the 19-item instrument described by Kindermann et al. (2024), with content selected and adapted for the study context. RDS itself used only the two organizational-source fields. The correlation describes convergence between the observed summaries, and the teacher aggregate provided an alternative H1 operationalization (Supplementary Figure S1 and Supplementary Table S10).

#### 2.3.2 Teacher-reported response indices

DSE comprised four T1 items with numeric responses from 1 to 7. Functional appraisal and digital stress comprised 10 and 8 T2 items, respectively, with responses from 1 to 5. Their T0 adjustment indices comprised the first eight appraisal and first six stress items represented in the T2 arrays. The overlapping indices differed in composition across waves and were used for baseline adjustment. DSE task themes drew on computer self-efficacy and teacher digital-competence research (Compeau and Higgins, 1995; Hatlevik and Hatlevik, 2018; Tondeur et al., 2023). Appraisal content was selected from workplace-AI attitudes research (Park et al., 2024), and stress content drew on digital-stress and occupational-technostress research (Hall et al., 2021; Funke et al., 2026; Ragu-Nathan et al., 2008). These sources document the conceptual and item-content lineage of the adaptations.

The adaptations connect the source concepts to PE teaching tasks. Functional appraisal combines perceived adaptability and information quality with personal utility, including confidence, accomplishment, and control. This personal-utility content is conceptually adjacent to efficacy beliefs. The stress items combine felt pressure, burden, or unease with appraised workload, learning effort, and disruption. Supplementary File S3 presents the Chinese field items and their English publication translations. The English version was prepared for publication rather than administered separately. The archive contains no documentation of an independent content-validation panel, cognitive interviewing, or cross-language validation. Response descriptions retain the recorded numeric ranges.

#### 2.3.3 Study-specific teacher-report AI-CTB score

The five AI-CTB items were adapted from the employee-AI collaboration measure developed by Kong et al. (2023), with employee and task referents tailored to PE teaching (Supplementary File S3). The items address reported AI involvement in decision making, prediction, problem solving or diagnosis, information evaluation, and recognition of classroom risks or improvement opportunities. The same items and 1–7 numeric response range were used at T0 and T3. Their arithmetic mean forms the study-specific teacher-reported practice score.

The T3 AI-CTB mean was standardized across the analytic cohort before analysis. Internal consistency was *α* = 0.852 at T3 and *α* = 0.878 at T0. The identical five-item set provided a source-matched baseline; longitudinal measurement invariance was not tested.

#### 2.3.4 Covariates

Models included the corresponding T0 score or overlapping response index, age, teaching experience, weekly PE hours, average class size, prior technology experience, region, institutional stage, sector, location, gender, educational qualification, and prior AI-related training. These variables represented differences in background, teaching workload, experience, and institutional setting. The primary specification separated institution means from teacher deviations for DSE and the focal baseline and T2 response scores. Other teacher-varying covariates entered with common within- and between-institution slopes. A *post hoc* full Mundlak specification relaxed this restriction for all teacher-varying numeric and categorical covariates. Table 3 summarizes the measures, sources, scoring, and descriptive measurement evidence.

**Table 3:** Measures, sources, scoring, and descriptive measurement evidence.

Observed variable/source	Role	Source	Function represented	Indicators/scoring	Evidence
RDS: institution-head source	Predictor component	Institution-head source	Reported institutional digitalization summary	Retained 1–7 source-summary field; standardized	M = 4.31 (SD = 1.30), *N* = 126
RDS: IT-lead source	Predictor component	Information-technology-lead source	Reported institutional digitalization summary	Retained 1–7 source-summary field; standardized	M = 4.14 (SD = 1.25), *N* = 126
Two-source RDS	Focal institution-level predictor	Institution-head and IT-lead sources	Observed organizational-source proxy; original component content unverified	Equal-weight standardized scores; restandardized	M = 0.066 (SD = 0.961) in 120 analytic institutions
Teacher-perceived digitalization conditions	Teacher questionnaire block; response-model factor	Teacher	Study-specific teacher-perceived digitalization responses	16 items; 1–7	*α* = 0.957; M = 4.158 (SD = 0.878)
DSE index (T1)	Cross-level interaction variable; response-model factor	Teacher	Confidence in digital teaching tasks; proposed personal resource	4 items; 1–7	*α* = 0.883; M = 4.097 (SD = 0.999)
Functional-appraisal index (T2)	Intermediate index in coefficient product; response-model factor	Teacher	Evaluation of AI adaptability, quality, and personal utility; not a job-resource measure	10 T2 items; 1–5; T0 covariate: 8 overlapping items	*α* = 0.924; M = 3.075 (SD = 0.680)
Digital-stress index (T2)	Intermediate index in coefficient product; response-model factor	Teacher	Perceived digitally related burden and strain; no separate exposure measure	8 T2 items; 1–5; T0 covariate: 6 overlapping items	*α* = 0.891; M = 2.582 (SD = 0.689)
Five-item, study-specific teacher-report AI-CTB score	Outcome; response-model factor	Teacher	Reported AI involvement in work tasks; not independent performance evidence	5 items; 1–7; corresponding T0 score	*α* = 0.852; M = 4.052 (SD = 0.972)

RDS, two-source reported digitalization proxy; DSE, digital self-efficacy; AI-CTB, five-item, study-specific teacher-reported AI involvement in teaching tasks. Appraisal captures evaluations of AI; digital stress combines perceived digital burden and strain. Alpha describes internal consistency of the teacher item blocks. RDS was constructed from archived summary fields rather than a reflective item-level scale, so no alpha coefficient is reported for it.

### 2.4 Statistical analysis

We examined the internal structure of the teacher-response measures using confirmatory factor analysis in R 4.6.1 and lavaan 0.6-21 with robust maximum likelihood (MLR; Rosseel, 2012). The five-factor model contained 43 indicators across T1–T3: perceived digitalization (16), DSE (4), appraisal (10), digital stress (8), and AI-CTB (5). Fit was assessed with the comparative fit index (CFI), Tucker–Lewis index (TLI), root mean square error of approximation (RMSEA) and its 90% interval, and standardized root mean square residual (SRMR). We examined model-based reliability, average variance extracted (AVE), and the heterotrait–monotrait ratio (HTMT) alongside these fit indices (Fornell and Larcker, 1981; Hu and Bentler, 1999; Henseler et al., 2015). Baseline-index reliability and fit were assessed separately (Dunn et al., 2014). Supplementary Sections S1.2 and S1.16 report the calculation details, alternative factor models, and cluster-corrected and ordinal sensitivities. RDS entered the regressions as an observed contextual proxy, separately from the teacher-response factor model.

We fitted observed-score ordinary least-squares regressions with within–between decomposition and institution-clustered inference in Python 3.12.13, using statsmodels 0.14.6, pandas 3.0.1, NumPy 2.3.5, and SciPy 1.18.0. RDS varies between institutions, whereas the teacher-response scores vary both between institutions and among colleagues. Distinguishing these comparisons separates institutional context from individual differences within the same workplace (Preacher et al., 2010). Each institution mean was the arithmetic mean of its retained teachers’ scores, and each teacher deviation was the difference from that mean. Institution means describe groups of sampled teachers; teacher-relative scores describe an individual’s position within the workplace. Teachers received equal regression weight, giving institutions with more retained teachers greater representation. The decomposition allows within- and between-institution associations to differ, while clustered inference accommodates dependence among teachers in the same institution. Random intercepts, random slopes, and multilevel latent variables were not estimated. The H1 model was:

$$\begin{array}{c} \begin{array}{l} \mathrm{AI}-{\mathrm{CTB}}_{T3,\mathrm{ij}}=\beta_{0}+c_{T}{\mathrm{RDS}}_{j}+\beta_{1W}\\ (\mathrm{AI}-{\mathrm{CTB}}_{T0,\mathrm{ij}}-\bar{\mathrm{AI}-{\mathrm{CTB}}_{T0,j}})+\beta_{1B}\bar{\mathrm{AI}-{\mathrm{CTB}}_{T0,j}} \end{array}\\ +\beta_{2W}({\mathrm{DSE}}_{T1,\mathrm{ij}}-\bar{{\mathrm{DSE}}_{T1,j}})+\beta_{2B}\bar{{\mathrm{DSE}}_{T1,j}}+X_{\mathrm{ij}}\gamma +Z_{j}\delta +\varepsilon_{\mathrm{ij}} \end{array}$$

Here, *i* indexes teachers and *j* institutions; the overbar denotes an institution mean, c_T_ is the RDS coefficient, X_ij_ and Z_j_ contain covariates, and ε_ij_ is the regression error. H1 includes institution means and teacher deviations for baseline AI-CTB and DSE, without the T2 response indices. Teacher scores were standardized across the 961-record cohort before decomposition, and RDS across the 126-institution source frame. Institution means and teacher deviations retained those scales without restandardization. Coefficients labeled *β* therefore use standardized observed-score scales rather than full multilevel standardization.

For H2 and H3, a is the RDS coefficient in the corresponding additive T2 appraisal or stress equation, adjusted for that index’s T0 components, DSE, and covariates. The coefficient b_B_ is the institution-mean T2 response coefficient in the shared T3 AI-CTB equation, which also includes both T2 teacher deviations, both T0 response indices at both levels, baseline AI-CTB, DSE, RDS, and covariates. The product a × b_B_ summarizes the proposed between-institution association sequence: H2 specifies a positive appraisal-related product, and H3 a negative stress-related product. Both component directions are reported, and their joint sampling uncertainty is retained (Preacher et al., 2010). These are associative estimands. The confounding assumptions required for causal mediation are unverified, and H1 and the products do not form a total/direct/indirect-effect decomposition.

H4a and H4b use separate T2 response equations containing RDS × teacher-relative DSE, all lower-order terms, and the nonfocal RDS × institution-mean DSE interaction. The focal coefficient describes how the RDS–response association varies with a teacher’s confidence relative to colleagues. Supplementary Section S1.16 provides the full model specifications, scale conventions, and estimand definitions.

Coefficient tables report finite-cluster-corrected sandwich standard errors with Student t inference and 119 cluster degrees of freedom (Cameron and Miller, 2015). For the primary H1–H4b family, we drew 5,000 bootstrap samples of 120 intact institutions with replacement (seed 20260728), using the same sample across equations in each draw to preserve dependence among estimates (Davison and Hinkley, 1997). We calculated pointwise percentile intervals and two-sided sign-proportion *p* values with a one-resample correction, then applied Holm adjustment across the five theory-guided hypotheses. Hypothesis decisions use the Holm-adjusted *p* values; intervals remain unadjusted. The study was not preregistered, and no timestamped analysis plan or *a priori* power analysis was available. Primary therefore identifies the central reported hypothesis family.

*Post hoc* one-way random-effects ANOVA summarized variance partitioning and the stability of sampled institution means. ICC(1) describes between-institution variation, and ICC(2) describes approximate mean stability under the one-way model and effective cluster size. These diagnostics concern the teacher aggregates. Institution-clustered inference and resampling accommodate dependence while retaining the observed means as measured.

Secondary analyses examined baseline and covariate specification, alternative estimators, source summaries, weighting and aggregation, item composition, educational stage, influence, and functional form. Conditional slopes, paired magnitude contrasts, and the Johnson–Neyman analysis were also *post hoc*. Supplementary Sections S1.4–S1.16 provide the full methods and results. The main Results present H1 specification dependence and the principal conditional-slope pattern because they are central to interpretation.

## 3 Results

### 3.1 Sample flow and measurement evidence

The cohort contained 961 teachers in 120 institutions, with 3–14 retained teachers per institution (M = 8.01, median = 8, IQR = 7–9). Tables 1–3 report record classifications, sample characteristics, and scores; Supplementary Tables S8–S9 provide level-specific correlations.

Retained and excluded records differed little on the three categorical fields available for all records: gender (Cramér’s V = 0.044), region (V = 0.021), and institutional stage (V = 0.040; Supplementary Table S14).

The five-factor MLR model yielded scaled χ^2^(850) = 2527.51, *p* < 0.001, CFI = 0.929, TLI = 0.924, RMSEA = 0.045, 90% CI [0.043, 0.047], and SRMR = 0.046. CFI and TLI fell below the conventional 0.95 benchmark, whereas the approximate-error indices indicated acceptable fit. Supplementary Table S2 reports the factor comparisons and alternative-estimator results. The cluster-corrected parameter covariance was rank deficient (120/139), restricting that solution to a global-fit sensitivity.

Across the five teacher-response blocks, α ranged from 0.852 to 0.957, model-based reliability from 0.857 to 0.958, and average variance extracted from 0.513 to 0.662. The maximum HTMT was 0.362. These estimates describe the internal structure and empirical separation of the response blocks (Supplementary Tables S3, S3A, and S17).

The *post hoc* ICC(1)/ICC(2) estimates were 0.270/0.748 for appraisal and 0.264/0.742 for digital stress. Institution means were less stable for DSE (ICC(2) = 0.380), baseline AI-CTB (0.260), and T3 AI-CTB (0.587). Supplementary Table S7 reports the full estimates.

### 3.2 Primary prospective association and specification dependence

H1 was not supported in the primary model (*β* = 0.073, 95% institution-cluster bootstrap CI [−0.063, 0.210], Holm-adjusted *p* = 0.302). Adding RDS increased descriptive *R*^2^ by 0.002. The coefficient varied with baseline and covariate specification: *β* was 0.114 in the teacher-baseline-score-only model, 0.097 after adding the institution-mean baseline, and 0.028 in the full Mundlak sensitivity. Only the simpler teacher-score specification had a bootstrap interval excluding zero. Thus, the positive estimate was sensitive to how pre-existing institutional differences were represented (Supplementary Tables S5, S5A, and S21).

In the additive T2 response equations, RDS was positively associated with institution-average appraisal (*β* = 0.197, cluster-robust 95% CI [0.033, 0.361]) and digital stress (*β* = 0.375, [0.268, 0.481]). In the shared outcome equation, institution-average appraisal was positively associated with later AI-CTB (*β* = 0.255, [0.089, 0.421]), and institution-average stress was negatively associated with it (*β* = −0.265, [−0.427, −0.103]). Table 4 presents the component coefficients and teacher-deviation associations.

**Table 4:** Regression coefficients from the H1–H3 models.

Model quantity	Association represented	*β*	Cluster SE	*p*	95% cluster-robust CI
H1 focal coefficient	RDS coefficient in baseline-adjusted T3 AI-CTB equation	0.073	0.067	0.277	[−0.060, 0.206]
Appraisal equation: between-institution RDS coefficient	RDS-institution-mean T2 appraisal association	0.197	0.083	0.019	[0.033, 0.361]
Stress equation: between-institution RDS coefficient	RDS-institution-mean T2 stress association	0.375	0.054	<0.001	[0.268, 0.481]
Outcome equation: appraisal-mean coefficient	Institution-mean appraisal association with T3 AI-CTB	0.255	0.084	0.003	[0.089, 0.421]
Outcome equation: appraisal-deviation coefficient	Teacher appraisal-deviation association with T3 AI-CTB	0.159	0.039	<0.001	[0.080, 0.237]
Outcome equation: stress-mean coefficient	Institution-mean stress association with T3 AI-CTB	−0.265	0.082	0.002	[−0.427, −0.103]
Outcome equation: stress-deviation coefficient	Teacher stress-deviation association with T3 AI-CTB	−0.085	0.041	0.040	[−0.166, −0.004]
Outcome equation: adjusted RDS coefficient	RDS coefficient in the shared intermediate-index-adjusted outcome equation	0.105	0.073	0.156	[−0.040, 0.250]

Rows derive from separate observed-score OLS equations with within–between decomposition. H1 excludes the T2 response indices; the shared H2/H3 outcome equation includes those indices and their T0 components. Table 5 reports products of the corresponding between-institution coefficients. Scores were standardized before decomposition, and components retained those scales. Coefficient sizes are interpreted within their respective equations. Confidence intervals and p values use institution-clustered t inference with 119 degrees of freedom; these component statistics are unadjusted.

### 3.3 Coefficient products and cross-level interactions

The appraisal coefficient product was 0.050 (pointwise 95% bootstrap CI [0.003, 0.109], raw *p* = 0.038, Holm-adjusted *p* = 0.114). Thus H2 did not meet the adjusted criterion despite its positive pointwise interval. The stress coefficient product was −0.099 (CI [−0.184, −0.033], raw *p* = 0.005, adjusted *p* = 0.021), supporting H3. Table 5 presents all five primary tests.

**Table 5:** Primary hypothesis tests.

Hypothesis	Estimand	Estimate	Pointwise 95% bootstrap CI	Raw p; Holm-adjusted *p*
H1	Adjusted prospective RDS association	0.073	[−0.063, 0.210]	0.302; 0.302
H2	Between-institution coefficient product involving appraisal index	0.050	[0.003, 0.109]	0.038; 0.114
H3	Between-institution coefficient product involving digital-stress index	−0.099	[−0.184, −0.033]	0.005; 0.021
H4a	RDS × teacher-relative DSE for appraisal	0.064	[−0.002, 0.132]	0.056; 0.114
H4b	RDS × teacher-relative DSE for stress	−0.101	[−0.168, −0.037]	0.003; 0.016

The five primary tests use 5,000 shared intact-institution bootstrap resamples. Intervals are pointwise percentiles; two-sided sign-proportion *p*-values use a one-resample correction. Holm adjustment covers H1, H2, H3, H4a, and H4b at *α* = 0.05. H3 and H4b meet the adjusted criterion; H1, H2, and H4a do not. The post hoc algebraic sum is reported in Supplementary Table S13.

The *post hoc* paired contrasts were inconclusive for differences in absolute magnitude between the appraisal and stress products and between their DSE interactions (Supplementary Table S13).

The RDS × teacher-relative DSE coefficient was 0.064 for appraisal (pointwise bootstrap CI [−0.002, 0.132], Holm-adjusted *p* = 0.114) and −0.101 for digital stress (CI [−0.168, −0.037], adjusted *p* = 0.016). H4b was supported; H4a was not. The stress association was shallower at higher relative DSE but remained positive at −1 empirical within-institution SD, the mean, and +1 SD: the corresponding slopes were 0.466, 0.374, and 0.282, and all pointwise intervals excluded zero (Figure 2; Supplementary Section S1.5).

**Figure 2:**
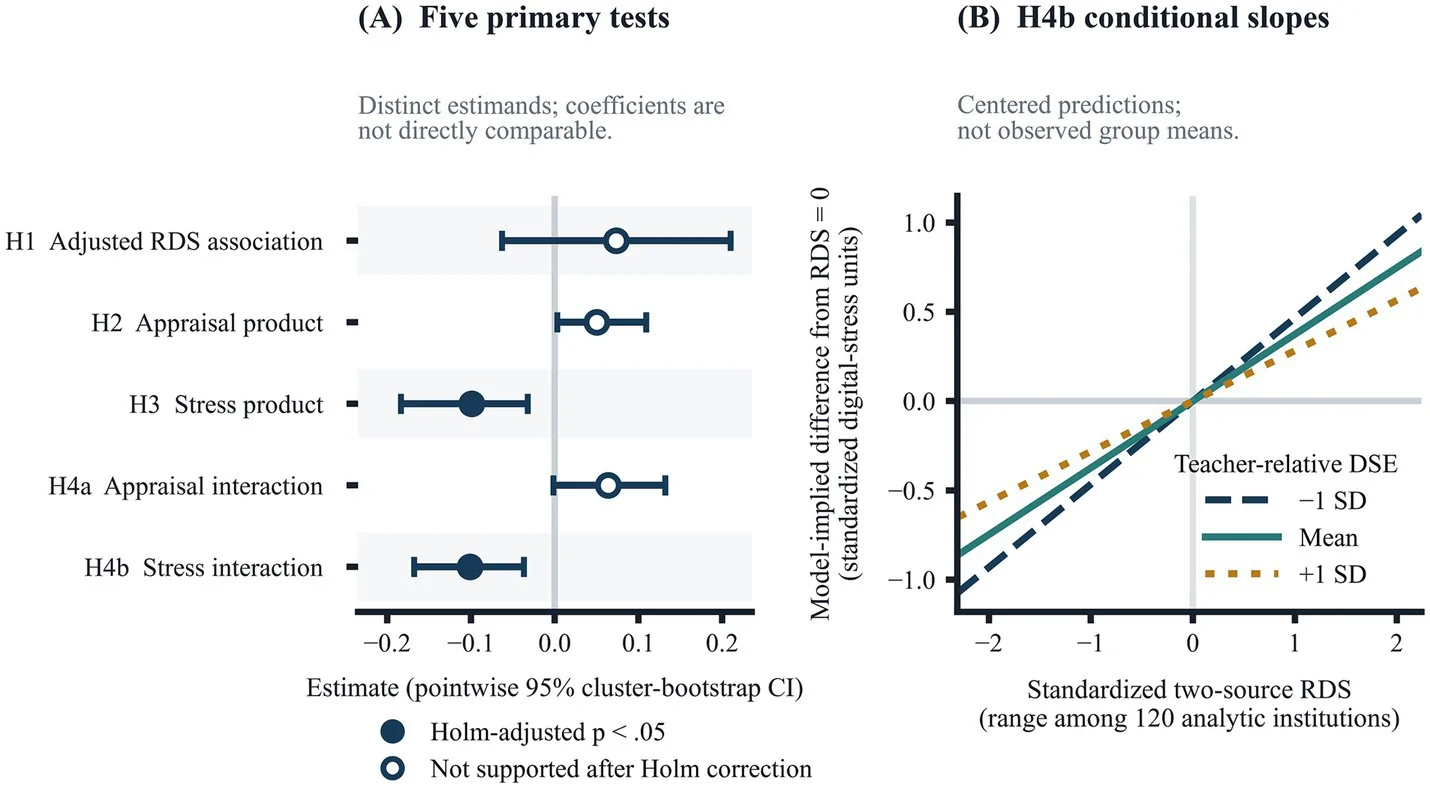
Primary estimates and conditional RDS–digital-stress associations. **(A)** Estimates and pointwise 95% institution-cluster bootstrap intervals. Filled markers indicate Holm-adjusted *p* < 0.05; open markers indicate hypotheses that did not meet the adjusted criterion. Each estimate is interpreted within its respective equation. **(B)** Centered predictions of standardized T2 digital stress relative to RDS = 0 at the mean and ±1 empirical within-institution SD of teacher-relative DSE (SD = 0.903). Slopes were 0.466 (95% bootstrap CI [0.329, 0.594]), 0.374 ([0.252, 0.486]), and 0.282 ([0.150, 0.410]), respectively. The conditional association was weaker at higher relative DSE and remained positive at all three probes. Supplementary Section S1.5 reports the exploratory Johnson–Neyman analysis.

### 3.4 Secondary analyses

H3 and H4b retained negative signs and met the adjusted criterion in the full Mundlak and matched-item five-test families. Other source, weighting, aggregation, and diagnostic checks used descriptive or explicitly unadjusted *post hoc* inference. Educational-stage contrasts were imprecise. Supplementary Tables S5–S5A, S10, S15, and S19–S27 report the complete secondary analyses, including H1 specification dependence.

## 4 Discussion

### 4.1 Principal findings

Perceived digital work pressure was central to the observed pattern. Institutions with higher RDS had higher average teacher-reported digital stress, which was associated with lower subsequent AI-CTB after baseline adjustment. The positive RDS–stress association was weaker among teachers with higher DSE relative to their colleagues. H3 and H4b met the multiplicity-adjusted criterion, whereas H1, H2, and H4a were not supported. This asymmetry in statistical support warrants attention to the stress-related findings, although paired comparisons did not establish differences in absolute coefficient magnitude. Together, the findings connect teachers’ experience of digital work with their reported AI-related task practice, complementing accounts centered on acceptance and confidence.

### 4.2 Institutional digitalization as a distal work context

RDS provides a broad organizational context for interpreting teachers’ reported AI practice. Its strong correspondence with the separate teacher-perceived digitalization aggregate indicates agreement between the available summaries. Work-design research offers a more differentiated account of that context by distinguishing infrastructure, support, autonomy, and workload, each with potentially different implications for practice (Wang et al., 2020; Parker and Grote, 2022). These distinctions help frame the organizational questions raised by the present findings; the archived RDS components leave the particular work arrangements involved unresolved.

H1 was not supported in the primary model: after accounting for baseline practice and analytic level, the estimate for RDS remained imprecise and its incremental contribution was small. One possible explanation is that digital systems and established teaching practices develop together. A broad context score would then overlap with existing institutional differences, while evaluations of specific tasks could capture aspects of later practice more directly. This interpretation makes the relationship between established work arrangements and subsequent AI use a useful focus for further study.

The sensitivity of H1 to baseline specification underscores the importance of those institutional differences. Including institution-average baseline practice distinguishes variation across workplaces from teachers’ own prior reports, and the further reduction under full covariate decomposition shows how the estimate depends on the representation of background differences. Future research could sharpen this comparison by measuring the work arrangements associated with both established and subsequent AI use.

### 4.3 Different evidence for functional appraisal and digital stress

Functional appraisal concerns what teachers believe AI can offer, whereas AI-CTB concerns its reported involvement in specific work tasks. Both appraisal-related component associations were positive, but their product did not meet the multiplicity-adjusted criterion. The appraisal-related sequence therefore remains inconclusive under the five-test criterion. Workplace-AI attitudes encompass perceived quality, adaptability, and personal utility (Park et al., 2024), and acceptance research links usefulness-related beliefs to intention and use (Venkatesh et al., 2003). These evaluations do not, by themselves, establish that AI outputs fit a lesson’s timing and information needs. A PE teacher can value movement analysis yet find device preparation or output checking difficult to accommodate while supervising an active class. Task fit offers a plausible explanation for why favorable evaluations need not consistently accompany greater AI involvement in teaching.

The stress-related sequence received clearer statistical support. One plausible interpretation is that perceived digital work pressure captures difficulties encountered in carrying out digitally supported tasks, whereas functional appraisal concerns what AI is judged to offer. The stress items refer to time pressure during device calibration, preparation and troubleshooting, reporting obligations, and disruption. Such experiences may be especially salient in PE, where attention devoted to digital systems must be coordinated with movement observation and immediate instructional decisions. This interpretation connects the findings to work-design and technostress research on the demands accompanying digital work (Ragu-Nathan et al., 2008; Tarafdar et al., 2019; Wang et al., 2020; Parker and Grote, 2022). Teacher studies have associated technostress with use intentions and well-being, while recent PE research has examined digital teaching demands alongside stress and burnout (Joo et al., 2016; Pagán-Garbín et al., 2024; Truța et al., 2023; Yang et al., 2026). The present study adds later, baseline-adjusted reports of task practice to this empirical picture.

The theoretical value of considering both experiences lies in separating perceived technological value from the perceived demands of incorporating technology into work. Separate measures of digital demands and task fit would help distinguish the proposed explanation from differences in the content of the adapted indices, including the combination of perceived requirements and strain in the stress score.

### 4.4 Variation in the RDS-digital-stress association by teacher-relative DSE

The positive RDS–stress association was weaker among teachers with higher DSE relative to their colleagues, yet remained positive at all conventional probe values. Confidence in digital diagnosis or assessment may make demanding tasks feel more manageable, consistent with DSE’s role as a personal resource. This interpretation concerns teachers’ perceived capacity to work within the available arrangements. The continuing positive association at the higher DSE probe suggests that confidence and the organization of digital work should be considered together. It motivates studies of self-efficacy development under different work requirements, rather than treating confidence development alone as a sufficient response to digital pressure.

H4a was not supported, leaving open how relative DSE relates to the association between institutional digitalization and functional appraisal. DSE concerns confidence in performing defined tasks, whereas appraisal combines judgments of AI’s quality and adaptability with personal benefits, including confidence and control. Efficacy beliefs could be relevant to perceived task difficulty without consistently strengthening evaluations across all these domains. This distinction offers a reason to examine the two associations separately. It complements work-design scholarship that considers employees’ capabilities within the organizational arrangements in which they are used (Parker and Grote, 2022).

### 4.5 Contribution to a JD-R-informed account of AI-supported work

The study applies JD-R principles to the organization of AI-supported teaching. Its contribution is to distinguish teachers’ evaluations of AI from their experience of digital work pressure when examining subsequent reported practice. This distinction makes an organizational question explicit: under which working conditions do potentially useful digital capabilities become manageable parts of professional activity? The supported stress-related product and RDS × teacher-relative DSE interaction direct attention to perceived pressure and the personal confidence associated with its variation. They complement acceptance-based accounts by connecting reported practice to the experience of carrying out digital work. A fuller test of JD-R theory would examine these relationships using separate measures of digital demands and job resources, alongside established occupational outcomes.

The institutional perspective also locates personal confidence within a shared workplace. RDS compares institutional contexts, while teacher-relative DSE compares colleagues within them. Keeping these comparisons distinct connects organizational arrangements with variation in teachers’ experience without reducing digitalization to individual acceptance.

### 4.6 Implications for organizational practice

The findings support including perceived digital work pressure alongside reported AI practice and confidence when assessing institutional implementation. Work-design and technostress research suggests several organizational conditions to examine: time for learning systems, duplicated reporting, access to reliable technical assistance, and teachers’ discretion over when digital tools enter instruction (Ragu-Nathan et al., 2008; Wang et al., 2020; Parker and Grote, 2022). These proposals draw on the broader literature and require evaluation in this setting. DSE development belongs within a wider organizational approach to workable digital conditions.

PE gives these proposals concrete task referents. Digital systems can offer movement information while preparation and output checking compete with the attention teachers devote to students during an active lesson. This potential tension, reflected in the study’s task content, could guide field studies of preparation time, reporting procedures, and access to assistance. Such studies could assess workload and independently recorded practice. Comparisons across educational stages and teaching subjects would clarify how these arrangements relate to the embodied, time-constrained character of PE; the present stage analyses were inconclusive.

### 4.7 Strengths, limitations, and future directions

The four separated measurement windows provide temporal ordering for the teacher-response blocks, and the same five AI-CTB items supply a source-matched baseline. RDS draws on organizational-source summaries separate from the teacher-response arrays. Within–between decomposition identifies the level of each comparison, institution resampling preserves dependence across equations, and Holm adjustment treats the five hypotheses as one testing family. Together with the reported model specifications and sensitivity analyses, these features make the observational analysis transparent and its calculations inspectable.

Selection is the first limitation. The cohort contains 65.2% of source records, and the decision rules for 202 exclusions were unavailable, as were wave-specific response denominators, sampling probabilities, and focal scores for excluded teachers. The small differences in the available demographics leave selection on stress or AI practice unresolved, so the findings apply directly to the 961 retained teachers. Age also differed between the two archived versions for 174 records. Omitting age changed no focal point estimate by more than 0.000097, but neither version could be authenticated from the available records (Supplementary Tables S20 and S28).

Measurement also limits the theoretical interpretation. The RDS archive lacks the original items, scoring documentation, verified respondent roles, and records of independent completion or timing. Its specific organizational content therefore remains uncertain. The teacher measures are contextual adaptations with evidence of internal consistency and factor structure; further evidence is needed to establish content validity, cross-language equivalence, longitudinal invariance, and independent behavioral validity. English items served as publication translations and were not separately administered. The digital-stress index combines perceived requirements and strain, without separate measures of job demands or resources. DSE captures confidence rather than demonstrated competence, and AI-CTB captures self-reported task practice rather than performance, engagement, burnout, teaching effectiveness, or independently verified safety outcomes. The rank-deficient cluster-corrected CFA further limits assessment of measurement across institutions.

Institution means and causal interpretation present further limitations. The means were based on 3–14 retained teachers, with ICC(2) ranging from 0.260 for baseline AI-CTB to 0.748 for appraisal among the focal teacher measures. Clustered inference accounts for dependence but leaves sampling and measurement error in these averages; a single reliability correction cannot determine the direction of bias in the multivariable models. Despite temporal separation and baseline adjustment, teacher reports remain susceptible to common-method variance and shared response tendencies at both the individual and institution-mean levels. Unmeasured institutional conditions also offer alternative explanations for the associations. The coefficient products consequently describe temporally ordered associations rather than causal mediation or a decomposition of a total effect, and the design does not estimate within-person change. The absence of verified prospective analysis prespecification is an additional limitation.

Future studies can address these limitations by separately measuring digital requirements, resources, and psychological responses and preserving complete recruitment and administration records. Repeated within-person observations could examine whether reporting, troubleshooting, and connectivity demands coincide with changes in pressure and task practice. Independent usage records and observations, collected under appropriate ethical and data-governance arrangements, would assess correspondence with self-reports. Larger samples within institutions and models that incorporate uncertainty in institution means would improve contextual estimates. Comparisons across educational stages and subjects could test the role of embodied, time-constrained tasks, while preregistered interventions or stronger quasi-experimental designs could evaluate organizational proposals alongside engagement, burnout, and performance.

### 4.8 Conclusion

Among the retained PE teachers, the stress-related coefficient product and the RDS × teacher-relative DSE interaction in the stress equation met the five-test criterion. The positive RDS–stress association was weaker at higher relative DSE and remained positive at conventional probe values. The adjusted RDS–practice association and appraisal-related hypotheses were not supported. These findings place teachers’ experience of digital work alongside their confidence and reported AI involvement in an organizational account of AI-supported teaching.

## Data Availability

Aggregate results and measurement documentation are provided in the article and its public Supplementary material. The underlying teacher-level and organizational-source records are not available for public release or private third-party sharing because the reviewed protocol restricted reporting to aggregate statistics and prohibited external sharing of the underlying records. The computational-verification package was supplied for editorial assessment and is not part of the public Supplementary material. Questions about the analyses and access restrictions may be directed to the corresponding author, Xiaoyun Wang (202599010062@swpu.edu.cn).
