# Supplementary material for: Institutional digitalization and digital stress: a four-wave observational study of teacher-reported AI-collaborative teaching in physical education
Source: Front Psychol. 2026 Oct 15;17:1973587. doi: 10.3389/fpsyg.2026.1973587 (PMC13716614; doi:10.3389/fpsyg.2026.1973587)
Supplement: Supplementary file 1 [file Data_Sheet_1.pdf]

Supplementary File S1

Measurement and supplementary analyses

This supplement documents the measurement evidence, primary hypothesis family, and post hoc sensitivity analyses for the retained four-wave cohort. RDS is an observed organizational-source proxy for reported institutional digitalization, constructed from two archived summary fields and kept separate from the teacher-questionnaire response model. The source labels, institution-head source and information-technology-lead source, follow the archived titles. The broader term institution-head accommodates both K-12 and higher education institutions. Supplementary File S3 documents the bilingual items and score construction. Section S1.16 gives the complete primary-model specification and defines the associational estimands.

S1.1 Variable construction

The two organizational-source summary fields were standardized separately across the 126 source institutions using population standard deviations, averaged with equal weights, and restandardized across the same source frame. The 120 institutions in the complete-record cohort inherited their corresponding RDS values. T0 and T3 AI-CTB scores were arithmetic means of the same five teacher-report items and were standardized separately by wave. Each focal teacher score was standardized across N = 961 before its arithmetic institution mean and teacher deviation were computed; these components retained their original standardized scale. These analysis-stage transformations are reproducible. The original source-item content, scoring decisions, contributor identities, independent completion, and actual source-specific completion times remain undocumented in the archive.

Table S1. Retained Variable Construction

Variable	Source	Construction	Analytic level
Two-source RDS	Institution-head and IT-lead source-summary fields	Equal mean of source-specific z scores; restandardized	Institution
DSE index	Teacher	Mean of 4 response positions; standardized; decomposed	Teacher/institution
Functional-appraisal index	Teacher	Mean of 10 T2 response positions; standardized; decomposed	Teacher/institution
Digital-stress index	Teacher	Mean of 8 T2 response positions; standardized; decomposed	Teacher/institution
AI-CTB score	Teacher	Mean of 5 T3 response positions; standardized; source-matched T0 score	Teacher/institution

S1.2 Questionnaire measurement evidence

The five-factor response model included 43 retained items from the T1-T3 teacher-questionnaire blocks and yielded CFI = 0.929, TLI = 0.924, RMSEA = 0.045, and SRMR = 0.046. Ordinary MLR describes the internal structure of these response arrays at a single level. The institution-cluster-corrected parameter covariance matrix had rank 120 of 139, limiting that analysis to a global-fit sensitivity rather than complete parameter-level validation. Ordinal WLSMV provides a further single-level sensitivity. Content validity, external behavioral validity, cross-language equivalence, multilevel measurement invariance, and longitudinal measurement invariance were not established by these analyses. The retained measures also combine aspects that JD-R theory distinguishes: the digital-stress bank covers appraised workload, learning effort, and disruption alongside pressure, burden, or unease, while functional appraisal captures evaluations of AI. Separate measures of digital job demands and job resources would be needed to distinguish work exposures from teachers' responses.

Table S2. Measurement-Model Fit

Model	Estimator	χ^2	df	CFI	TLI	RMSEA [90% CI]	SRMR
Five-factor questionnaire model	MLR	2527.51	850	0.929	0.924	0.045 [0.043, 0.047]	0.046
Five-factor questionnaire model, institution-cluster corrected	MLR with institution-cluster correction	2532.41	850	0.932	0.928	0.045 [0.043, 0.047]	0.046
Four-factor model pooling DSE and AI-CTB	MLR	4367.69	854	0.850	0.842	0.065 [0.064, 0.067]	0.072
One-factor questionnaire model	MLR	13727.27	860	0.452	0.424	0.125 [0.123, 0.127]	0.165
Five-factor questionnaire model, ordinal WLSMV	WLSMV	3320.12	850	0.961	0.958	0.055 [0.053, 0.057]	0.049

Table S3. Reliability and Internal-Structure Summary

Construct	Response positions	Model-based reliability	AVE	Loading range
Teacher-perceived digitalization	16	0.958	0.589	0.615-0.854
DSE	4	0.886	0.662	0.723-0.894
Appraisal	10	0.924	0.551	0.666-0.797
DigitalStress	8	0.893	0.513	0.596-0.827
AI-CTB	5	0.857	0.553	0.540-0.885

Model-based reliability was computed as $(\Sigma\lambda)^2/[(\Sigma\lambda)^2 + \Sigma\theta]$ from the standardized loadings for each factor in the five-factor CFA, assuming uncorrelated item residuals. Under this specification, the formula is algebraically identical to the conventional composite-reliability expression, so no separate ω estimate is reported. The largest HTMT ratio was 0.362, between the two T1 blocks measuring teacher-perceived digitalization conditions and DSE. The same-wave T2 functional-appraisal-digital-stress HTMT was 0.101. Table S3A gives the complete pairwise matrix. These values describe empirical separation of the retained response blocks; content validity and longitudinal discriminant validity require separate evidence.

Table S3A. Complete Pairwise HTMT Matrix

Block	T1 digitalization	T1 DSE	T2 appraisal	T2 stress	T3 AI-CTB
T1 digitalization	-	0.362	0.280	0.131	0.228
T1 DSE	0.362	-	0.322	0.226	0.311
T2 appraisal	0.280	0.322	-	0.101	0.360
T2 stress	0.131	0.226	0.101	-	0.191
T3 AI-CTB	0.228	0.311	0.360	0.191	-

Note. The model combines response blocks administered at different waves. The maximum value (0.362) is between two same-wave T1 blocks; the T2 appraisal-stress value is 0.101.

S1.3 Hypothesis-family bootstrap

The theory-guided primary family comprises H1, H2, H3, H4a, and H4b. Primary denotes the focal family reported here: the study was not preregistered, and no timestamped analysis plan was available. Each of 5,000 bootstrap draws sampled 120 intact institutions with replacement (seed 20260728), using the same sampled institutions across equations. Pointwise percentile intervals and two-sided sign-proportion p values were calculated from these draws, followed by Holm adjustment across the five hypotheses. H3 and H4b met the adjusted criterion; H1, H2, and H4a did not. H2's pointwise interval excludes zero, but its Holm-adjusted p value falls above the familywise threshold. Section S1.16 defines the resampling procedure and p-value calculation.

Table S4. Hypothesis Family

Hypothesis	Estimate	95% CI	Raw p	Holm p	Supported
H1	0.073	[-0.063, 0.210]	0.302	0.302	No
H2	0.050	[0.003, 0.109]	0.038	0.114	No
H3	-0.099	[-0.184, -0.033]	0.005	0.021	Yes
H4a	0.064	[-0.002, 0.132]	0.056	0.114	No
H4b	-0.101	[-0.168, -0.037]	0.003	0.016	Yes

S1.4 H1 baseline-level and influence checks

The primary H1 model includes separate institution means and teacher deviations for baseline AI-CTB and DSE. In a post hoc specification check, the simpler teacher-score model yielded a positive unadjusted result, which was not reproduced after institution-mean baseline adjustment or in the primary within-between model. The full Mundlak sensitivity in Section S1.11 further examines institution-level covariate differences. H1 remains unsupported in the primary family; the simpler-model result describes its sensitivity to specification.

Table S5. H1 Baseline-Level Sensitivity

Specification	β	Cluster CI	Bootstrap CI	Bootstrap p
teacher level baseline only	0.114	[0.032, 0.196]	[0.030, 0.201]	0.010
institution mean baseline added	0.097	[-0.017, 0.211]	[-0.023, 0.213]	0.112
primary full within between	0.073	[-0.060, 0.206]	[-0.063, 0.210]	0.302

Table S5A. Additional H1 Estimator and Influence Checks

Model/check	β or range	SE	p	95% CI	Interpretation
Unadjusted	.252	.044	<.001	[.166, .339]	Attenuated after adjustment
Generalized estimating equations	.089	.063	.158	[-.034, .211]	Comparable to the primary estimate
Cook's-distance deletion	.080	.059	.181	[-.038, .197]	52 teachers removed
Leave-one-institution-out	.049 to .107	N/A	maximum p = .454	Not reported	No sign reversals across 120 refits

Note. These post hoc checks are descriptive and do not replace the five-test Holm family. The GEE row uses a robust sandwich interval. Leave-one-institution-out reports the coefficient range and maximum p value across 120 refits.

S1.5 H4b conditional RDS-digital-stress slopes

These post hoc simple slopes describe the fitted H4b interaction at the mean and ±1 empirical SD of teacher-relative DSE (SD = 0.903), with institution-mean DSE fixed at zero on the standardized scale. Higher teacher-relative DSE corresponded to a shallower positive RDS-digital-stress association, which remained positive at all three probe values. The estimates describe conditional associations rather than the effects of increasing DSE or removing organizational digital pressures.

Table S6. H4b Simple Slopes

DSE condition	Slope	Cluster CI	Bootstrap CI
DSE -1 within-institution SD	0.466	[0.340, 0.591]	[0.329, 0.594]
DSE at institution mean	0.374	[0.267, 0.481]	[0.252, 0.486]
DSE +1 within-institution SD	0.282	[0.161, 0.403]	[0.150, 0.410]

Post hoc cluster-robust Johnson-Neyman inference placed the first boundary within the observed DSE range at 2.008 pooled-standardized units, equivalent to 2.22 empirical within-institution SDs. The conditional slope was distinguishable from zero below this boundary but not between the boundary and the observed maximum. Only 8 of 961 teachers (0.83%) were above the boundary, so the sparse tail provides little basis for interpreting it as a threshold at which DSE eliminates or reverses the association.

S1.6 Variance partitioning

Post hoc variance partitioning used one-way random-effects ANOVA. ICC(1) describes the between-institution share of response variance, and ANOVA-based ICC(2) approximates the stability of sampled institution means at the effective cluster size; these estimates were obtained from ANOVA rather than fitted null random-intercept models. Retained institution sizes ranged from 3 to 14 teachers, with a mean of 8.01, median of 8, and interquartile range of 7-9. The means summarize sampled teachers' responses, with contextual interpretation constrained by their variable reliability and the small, unequal cluster sizes. Shared organizational climate and RDS reliability require separate evidence. Measurement error in the teacher means remains after ICC(2) estimation and intact-cluster bootstrapping.

Table S7. Intraclass Correlations

Variable	ICC(1)	ICC(2)	Effective cluster size	Institutions
T0 AI-CTB	0.042	0.260	8.00	120
T1 DSE	0.071	0.380	8.00	120
T2 appraisal	0.270	0.748	8.00	120
T2 digital stress	0.264	0.742	8.00	120
T3 AI-CTB	0.151	0.587	8.00	120
Teacher-perceived digitalization conditions	0.293	0.768	8.00	120

S1.7 Level-specific correlations and sensitivity to predictor definition and educational stage

Within-institution correlations use teacher deviations from institution means (N = 961). Between-institution correlations use institution means (J = 120).

Table S8. Within-Institution Correlations

Variable	1	2	3	4	5	6	7	8
1. Teacher-perceived digitalization conditions	1.000	0.231	0.162	0.054	0.169	0.141	-0.004	0.077
2. DSE	0.231	1.000	0.256	-0.011	0.172	0.232	-0.278	0.222
3. T0 appraisal	0.162	0.256	1.000	-0.038	0.264	0.459	-0.180	0.188
4. T0 digital stress	0.054	-0.011	-0.038	1.000	-0.088	-0.022	0.320	-0.116
5. T0 AI-CTB	0.169	0.172	0.264	-0.088	1.000	0.160	-0.125	0.321
6. T2 appraisal	0.141	0.232	0.459	-0.022	0.160	1.000	-0.189	0.242
7. T2 digital stress	-0.004	-0.278	-0.180	0.320	-0.125	-0.189	1.000	-0.201
8. T3 AI-CTB	0.077	0.222	0.188	-0.116	0.321	0.242	-0.201	1.000

Table S9. Between-Institution Correlations

Variable	1	2	3	4	5	6	7	8	9	10	11
1. Two-source RDS	1.000	0.949	0.950	0.881	0.576	0.560	0.227	0.563	0.552	0.365	0.493
2. Institution-head-source summary	0.949	1.000	0.804	0.838	0.535	0.501	0.226	0.521	0.514	0.371	0.468
3. IT-lead-source summary	0.950	0.804	1.000	0.836	0.560	0.562	0.205	0.548	0.535	0.322	0.468
4. Teacher-perceived digitalization conditions mean	0.881	0.838	0.836	1.000	0.603	0.567	0.229	0.558	0.490	0.310	0.509
5. DSE mean	0.576	0.535	0.560	0.603	1.000	0.552	0.178	0.413	0.491	-0.040	0.452
6. T0 appraisal mean	0.560	0.501	0.562	0.567	0.552	1.000	0.031	0.517	0.417	0.129	0.360
7. T0 digital stress mean	0.227	0.226	0.205	0.229	0.178	0.031	1.000	0.188	0.056	0.392	0.142
8. T0 AI-CTB mean	0.563	0.521	0.548	0.558	0.413	0.517	0.188	1.000	0.299	0.231	0.407
9. T2 appraisal mean	0.552	0.514	0.535	0.490	0.491	0.417	0.056	0.299	1.000	0.097	0.522
10. T2 digital stress mean	0.365	0.371	0.322	0.310	-0.040	0.129	0.392	0.231	0.097	1.000	-0.106
11. T3 AI-CTB mean	0.493	0.468	0.468	0.509	0.452	0.360	0.142	0.407	0.522	-0.106	1.000

Note. RDS and the teacher-perceived institution mean showed strong observed convergence (r = 0.881). ICC(2) in Table S7 summarizes the expected stability of the teacher mean under the one-way model; without source-specific measurement models and verified independent errors, it does not identify a latent correlation between the two contextual summaries.

Table S10. Sensitivity to Predictor Definition and Educational Stage

Sensitivity	Estimate	Cluster CI	Bootstrap CI	Bootstrap p
two-source RDS (primary)	0.073	[-0.060, 0.206]	[-0.063, 0.210]	0.302
institution-head-source summary only	0.076	[-0.035, 0.188]	[-0.044, 0.197]	0.214
Information-technology-lead-source summary only	0.046	[-0.086, 0.178]	[-0.088, 0.182]	0.495
teacher-perceived institution mean	0.096	[-0.018, 0.210]	[-0.031, 0.214]	0.129
K-12 slope	0.102	[-0.046, 0.250]	[-0.051, 0.258]	0.197
Higher education slope	-0.014	[-0.177, 0.148]	[-0.194, 0.175]	0.915
Higher education minus K-12	-0.116	[-0.290, 0.057]	[-0.309, 0.081]	0.251

Note. The teacher-perceived institution mean provides an alternative H1 operationalization, not an independent replication. Given its strong correlation with RDS, similar estimates are expected, but the adjusted coefficients are not algebraically constrained to coincide.

Figure S1. Institution-level convergence between RDS and teacher-perceived digitalization conditions

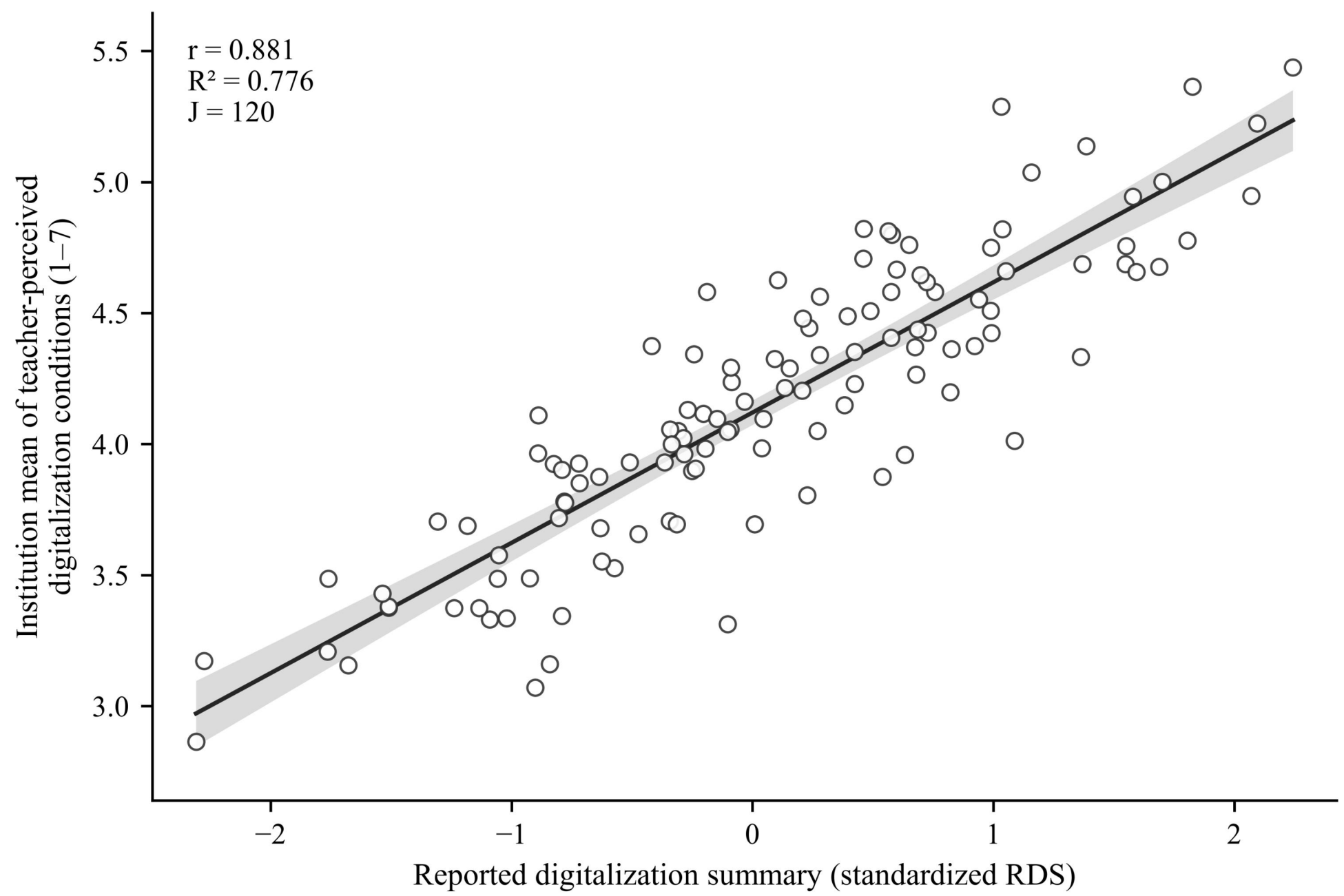

Note. Points represent the 120 institutions in the analytic cohort. The line is the ordinary least-squares fit with a 95% confidence band. RDS was constructed from the two organizational-source summary fields; the vertical axis is the arithmetic institution mean of the separate 16-item teacher block. Teachers have equal weight within each mean and in the primary regressions. Larger retained institutions therefore contribute more equally weighted teacher observations to the regressions.

S1.8 Unified-baseline and additional robustness analyses

The shared T3 AI-CTB equation for H2 and H3 includes within- and between-institution T0 appraisal and digital-stress scores, the corresponding T2 score components, baseline AI-CTB components, DSE components, RDS, and the covariates listed in Section S1.16. Tables S4 and S12 present the same primary five-hypothesis family; Table S12 repeats the results beside the full outcome equation for traceability. Follow-up contrasts, selection descriptions, institutional-stage analyses, and item-composition sensitivities are post hoc.

Table S11. Unified-Baseline AI-CTB Outcome Equation

Outcome-equation term	Estimate	Cluster SE	95% CI	p
Intermediate-index- and baseline-adjusted RDS coefficient	0.105	0.073	[-0.040, 0.250]	0.156
T2 appraisal within-institution	0.159	0.039	[0.080, 0.237]	< 0.001
T2 appraisal institution mean	0.255	0.084	[0.089, 0.421]	0.003
T2 stress within-institution	-0.085	0.041	[-0.166, -0.004]	0.040
T2 stress institution mean	-0.265	0.082	[-0.427, -0.103]	0.002
T0 appraisal within-institution	-0.003	0.035	[-0.073, 0.067]	0.929
T0 appraisal institution mean	-0.019	0.112	[-0.240, 0.203]	0.868
T0 stress within-institution	-0.064	0.036	[-0.136, 0.008]	0.082
T0 stress institution mean	0.191	0.101	[-0.009, 0.391]	0.061
T1 DSE within-institution	0.097	0.032	[0.033, 0.162]	0.003
T1 DSE institution mean	0.003	0.115	[-0.224, 0.231]	0.978
T0 AI-CTB within-institution	0.234	0.034	[0.168, 0.301]	< 0.001
T0 AI-CTB institution mean	0.335	0.121	[0.097, 0.574]	0.006

Note. N = 961 teachers in 120 institutions. With continuous controls standardized, the design matrix was full rank (34/34) and its condition number was 18.12. In unscaled units the condition number was 972.75; rescaling left fitted values and focal estimates unchanged. Coefficients use standardized observed scores, but decomposed components were not restandardized. The RDS coefficient of 0.105 is conditional on the included T2 indices and baseline terms; it is not an identified direct effect. Coefficient intervals and p values use finite-cluster-corrected sandwich inference with 119 degrees of freedom and are distinct from the primary bootstrap family.

Table S12. Five-Hypothesis Family Under the Unified-Baseline Specification

Quantity	Estimate	95% bootstrap CI	Raw p	Holm p	Supported
H1	0.073	[-0.063, 0.210]	0.302	0.302	No
H2	0.050	[0.003, 0.109]	0.038	0.114	No
H3	-0.099	[-0.184, -0.033]	0.005	0.021	Yes
H4a	0.064	[-0.002, 0.132]	0.056	0.114	No
H4b	-0.101	[-0.168, -0.037]	0.003	0.016	Yes

Table S13. Paired-Bootstrap Contrasts and Algebraic Sum of Coefficient Products

Post hoc quantity	Point estimate	95% bootstrap CI	p
H3 - H2 (signed)	-0.150	[-0.243, -0.062]	0.002
|H3| - |H2|	0.049	[-0.035, 0.153]	0.253
|H4b| - |H4a|	0.038	[-0.058, 0.131]	0.432
H2 + H3	-0.049	[-0.155, 0.036]	0.254

Note. These post hoc quantities were not added to the five-hypothesis family. A signed difference can be nonzero because estimates point in opposite directions even when their absolute magnitudes do not differ. Neither absolute-magnitude contrast was conclusive, so clearer adjusted support for H3 and H4b does not establish larger coefficients than H2 and H4a. H2 + H3 is an algebraic sum of descriptive coefficient products, not a total indirect effect or a proportion mediated.

Table S14. Limited Retained-Versus-Excluded Tracking Comparison

Tracking field	Retained n	Excluded n	Test	p	Cramer's V
Gender	961	513	$\chi^2(1) = 2.90$	0.089	0.044
Region	961	513	$\chi^2(3) = 0.67$	0.880	0.021
Institutional stage	961	513	$\chi^2(3) = 2.38$	0.497	0.040

Note. The tracking file contained these three fields for all 1,474 records. Age was excluded from this comparison because 174 of 961 retained values differed from the analysis file; neither version could be authenticated from the migrated archive. The cohort includes 961 of the 1,474 tracked teacher records (65.2%), drawn from 120 of the 126 source institutions. The other 513 records were assigned mutually exclusive archived categories: no T1 response (77), no T2 response (96), no T3 response (84), invalid matching code (54), historical quality-control label (59), and upstream eligibility or record-completeness exclusion (143). These counts describe retained classifications rather than a reconstructed sequential attrition history. Executable decision rules were unavailable for the last two categories, covering 202 records. Missing denominators and response histories prevent estimation of wave-specific response rates and selection probabilities. Small differences in the available tracking fields cannot rule out selection on digitalization,

stress, AI practice, or unavailable covariates; the findings apply directly to the complete-record cohort, without establishing national or institution-wide representativeness.

Table S15. Pooled K-12-Versus-Higher-Education Interaction Sensitivity

Stage-heterogeneity quantity	Difference (HE - K-12)	95% bootstrap CI	p
H3 coefficient-product association	-0.110	[-0.346, 0.074]	0.255
H4b interaction	-0.114	[-0.258, 0.045]	0.150

Note. K-12 comprised 709 teachers in 89 institutions; higher education comprised 252 teachers in 31 institutions. Intervals are imprecise heterogeneity tests and are not equivalence intervals. The pooled bootstrap design was full rank in all 5,000 resamples.

Table S16. H4b leave-one-institution-out influence

Specification	Point estimate	95% bootstrap CI	Bootstrap p	Deterministic range	Sign reversals
Two-source RDS (primary reference)	-0.101	[-0.168, -0.037]	0.003	Not applicable	Not applicable
Leave-one-institution-out	-0.101	Not applicable	Not applicable	[-0.116, -0.088]	0/120

Note. The first row reproduces the primary five-hypothesis-family estimate and its institution-cluster bootstrap interval and p value. The second row reports the deterministic leave-one-institution-out coefficient range and sign-reversal count. Source-specific, weighting, and leave-one-teacher-out estimates are reported once under the shared institution-resampling plan in Tables S22A and S22B.

Table S17. Baseline-Index Reliability and Single-Factor Fit

Baseline index	Response positions	α	ω	Loading range	CFI / TLI	RMSEA / SRMR
T0 functional-appraisal index	8	0.909	0.911	0.615 to 0.818	0.950 / 0.930	0.096 / 0.031
T0 digital-stress index	6	0.870	0.872	0.637 to 0.810	0.975 / 0.958	0.074 / 0.024

Note. Reliability and fit characterize the retained response arrays. Assessing content validity additionally requires evidence that the administered items correspond to the intended constructs. The appraisal model's RMSEA was 0.096, alongside CFI = 0.950 and SRMR = 0.031.

Table S18. Single-Level K-12-Versus-Higher-Education Equal-Loading Analysis

Model	Scaled χ^2	df	CFI	TLI	RMSEA [90% CI]	SRMR	ΔCFI	ΔRMSEA
Configural	3591.91	1700	0.922	0.917	0.048 [0.046, 0.050]	0.052	0.0000	0.0000
Metric/loadings equal	3621.93	1738	0.922	0.919	0.047 [0.045, 0.050]	0.053	0.0003	-0.0006

Note. Higher education: 252 teachers in 31 institutions; K-12: 709 teachers in 89 institutions. Robust metric-versus-configural difference: $\Delta\chi^2(38) = 26.04$, $p = 0.929$. This post hoc comparison concerns single-level loading equality across institutional stages. It does not test RDS, scalar invariance, multilevel invariance, longitudinal invariance, or equivalent regression coefficients.

S1.9 Item-composition-matched sensitivity

This post hoc check restricted the T2 appraisal and digital-stress indices to the eight and six numbered response positions, respectively, represented in the T0 arrays. The intermediate-index equations, unified outcome equation, within-between decomposition, covariates, N = 961 sample, 120-institution clustering, 5,000 resamples, and seed 20260728 were otherwise unchanged. All models and bootstrap refits were full rank.

Table S19. Item-Composition-Matched H2-H3 Sensitivity

Quantity	Estimate	95% bootstrap CI	Raw p	Sensitivity Holm p	Conclusion
H2, matched positions	.050	[.002, .108]	.040	.121	Not supported
H3, matched positions	-.102	[-.184, -.036]	.003	.014	Supported
Matched minus primary H2	-.0003	[-.0067, .0049]	.857	Not applicable	Negligible difference
Matched minus primary H3	-.0026	[-.0140, .0100]	.682	Not applicable	Negligible difference

Note. When the matched-position H2 and H3 estimates replaced their primary counterparts in the same five-test Holm family, the adjusted p values were .121 for H2, .014 for H3, .121 for H4a, .014 for H4b, and .302 for H1. The corresponding hypothesis decisions were unchanged. The matched T2 appraisal and stress subsets had α = .904 and .848 and correlated with the full T2 indices at r = .988 and .981, respectively. This analysis evaluates sensitivity to index composition; it is not a longitudinal measurement-invariance test.

S1.10 Control-collinearity sensitivity

Age and teaching experience were strongly correlated (r = 0.921). Standardizing all five continuous controls left H1-H4b unchanged. Omitting age or teaching experience separately changed no focal point estimate by more than 0.00033; the maximum change after omitting age was .000097. These checks assess point-estimate stability. Authentication of the age versions and a new bootstrap evaluation of the five-hypothesis family remain outside their scope.

Table S20. Control-collinearity sensitivity

Specification	H1	H2	H3	H4a	H4b	Largest |Δ|
All controls	.073198	.050193	-.099328	.063638	-.101467	Reference
Age omitted	.073101	.050175	-.099389	.063662	-.101477	.000097
Teaching experience omitted	.072899	.050038	-.099654	.063642	-.101287	.000326

Note. Variance-inflation factors were 7.37 for age and 7.19 for teaching experience in the unified outcome model. These are point-estimate sensitivities; primary bootstrap inference remains the family reported in Table S12.

S1.11 Full Mundlak covariate sensitivity

A post hoc full Mundlak sensitivity decomposed every teacher-varying numeric and categorical covariate into an institution mean and a teacher deviation. It used the same 5,000 institution-cluster bootstrap draws and five-hypothesis Holm family. The analysis evaluates sensitivity to the covariate-level assumptions while retaining the primary model as the reporting reference.

Table S21. Full Mundlak five-hypothesis family

Hypothesis	Estimate	Pointwise 95% CI	Raw p	Holm p	Decision at .05
H1	.028	[-.147, .185]	.733	.733	Not supported
H2	.055	[-.001, .131]	.057	.172	Not supported
H3	-.093	[-.186, -.023]	.008	.034	Supported
H4a	.062	[-.003, .129]	.065	.172	Not supported
H4b	-.104	[-.171, -.040]	.003	.014	Supported

Note. All six design matrices used by the hypothesis family were full rank in every bootstrap draw. H3 and H4b remained below zero with Holm-adjusted $p < .05$. The H1 point estimate decreased from .073 to .028, the largest descriptive shift in the family; no formal between-specification difference test is implied.

S1.12 Weighting and aggregation sensitivity

These checks examine unequal institution size and the reuse of teacher observations in calculating institution means. Each changes the weighting or aggregation of observations. Their intervals are therefore descriptive post hoc comparisons outside the primary Holm family.

Table S22A. Full-family weighting sensitivities

Specification	H1 [95% CI]	H2 [95% CI]	H3 [95% CI]	H4a [95% CI]	H4b [95% CI]
Primary composite-RDS reference	.073 [-.063, .210]	.050 [.003, .109]	-.099 [-.184, -.033]	.064 [-.002, .132]	-.101 [-.168, -.037]
Equal total teacher weight per institution	.091 [-.040, .213]	.049 [.004, .111]	-.108 [-.190, -.040]	.052 [-.009, .117]	-.091 [-.160, -.027]
Leave-one-teacher-out institution means	.074 [-.062, .211]	.048 [.001, .104]	-.097 [-.181, -.030]	.041 [-.024, .113]	-.087 [-.154, -.018]

Note. Intervals are pointwise percentiles from 5,000 shared institution-resampling draws. All within-draw paired differences between the weighting alternatives and the primary reference included zero. The alternatives estimate related but nonidentical weighted quantities; no multiplicity adjustment was applied.

Table S22B. Full-family source-summary sensitivities

RDS source	H1 [95% CI]	H2 [95% CI]	H3 [95% CI]	H4a [95% CI]	H4b [95% CI]
Institution-head summary	.076 [-.044, .197]	.042 [.001, .095]	-.092 [-.163, -.031]	.058 [-.010, .125]	-.114 [-.182, -.052]
Information-technology-lead summary	.046 [-.088, .182]	.046 [.0001, .104]	-.074 [-.150, -.019]	.061 [-.005, .129]	-.078 [-.145, -.012]

Note. Each source summary replaced the composite RDS throughout all equations. Paired-draw contrasts with the composite reference included zero except that the information-technology-lead estimates were less negative for H3 by .025, 95% CI [.001, .056], and for H4b by .024, 95% CI [.005, .045]. These unadjusted contrasts are descriptive and do not establish source-summary differences.

Table S22C. Collapsed institution-level aggregation check

Specification	H2 [95% CI]	H3 [95% CI]
Collapsed 120-institution model	.044 [-.004, .118]	-.104 [-.189, -.030]

Note. H2 crosses zero under this aggregation, whereas H3 remains below zero. The model evaluates a different analytical unit and weighting scheme as a sensitivity to the primary teacher-level analysis.

S1.13 Rank and institution-influence diagnostics

Table S23. Full Mundlak design-matrix diagnostics

Model	Rows	Columns	Rank	Condition number	NumPy-statsmodels max coefficient difference
H1	961	36	36	54.73	1.50e-15
Appraisal	961	36	36	54.56	3.44e-15
Stress	961	36	36	54.63	4.22e-15
Outcome	961	44	44	55.57	2.94e-15
H4a	961	38	38	55.99	5.66e-15
H4b	961	38	38	56.10	1.11e-15

Table S24. Leave-one-institution-out influence across the hypothesis family

Quantity	Full sample	Minimum	Maximum	Maximum |change|	Sign reversals
H1	.073	.049	.107	.034	0/120
H2	.050	.042	.059	.009	0/120
H3	-.099	-.121	-.086	.022	0/120
H4a	.064	.046	.073	.018	0/120
H4b	-.101	-.116	-.088	.015	0/120

Note. Rank and numerical agreement do not establish substantive model validity. The influence range for H1 is small in absolute terms but does not resolve its broader specification dependence.

S1.14 Functional-form, wild-cluster, and residual diagnostics

Table S25A. Quadratic main-effect checks

Model	Focal linear-term estimate in augmented model (p)	Added term	Added estimate (p)
H1	.077 (.241)	RDS squared	-.038 (.190)
Appraisal equation	.197 (.017)	RDS squared	-.049 (.294)
Stress equation	.374 (<.001)	RDS squared	.011 (.766)

Note. The p values in Table S25A use finite-cluster-corrected ordinary least-squares inference and are descriptive.

Table S25B. Quadratic-in-RDS cross-level interaction checks

Outcome equation	Term	Estimate	Pointwise 95% bootstrap CI	Sign-proportion p
Functional appraisal (H4a)	Linear RDS x DSE deviation	.064	[-.004, .135]	.060
Functional appraisal (H4a)	RDS squared x DSE deviation	-.012	[-.055, .042]	.605
Digital stress (H4b)	Linear RDS x DSE deviation	-.101	[-.170, -.036]	.004
Digital stress (H4b)	RDS squared x DSE deviation	-.022	[-.067, .033]	.413

Note. Intervals and p values use the same 5,000 shared institution-resampling draws as the balanced sensitivity suite. Each augmented equation included RDS, RDS squared, within- and between-institution DSE, and both linear and quadratic RDS-by-DSE terms. No multiplicity adjustment was applied. Complete coefficients and paired differences from the primary linear models were supplied in the computational-verification materials for editorial review.

Table S26. Restricted-null wild-cluster bootstrap checks

Quantity	Observed cluster t	Wild-cluster p	Draws	Weights
H1	1.092	.326	9,999	Rademacher
H4a	1.884	.077	9,999	Rademacher
H4b	-2.946	.008	9,999	Rademacher

Note. The restricted-null bootstrap-t omitted the tested term, fit the null OLS model, multiplied restricted residuals by institution-level Rademacher weights, and recomputed the finite-cluster-corrected CR1 t statistic in the full model. The random-number seed was 20260745, and p values use (exceedances + 1)/(draws + 1).

Table S27. Aggregate residual and leverage summaries

Model	Max individual hat value	Max institution hat-trace	Max |OLS externally studentized residual|	% |OLS externally studentized| > 3
H1	.077	.414	3.13	.21
Appraisal	.079	.449	4.39	.62
Stress	.075	.426	4.06	.62
Outcome	.089	.573	3.11	.31
H4a	.089	.473	4.49	.62
H4b	.091	.467	3.79	.52

Note. Quadratic-term and wild-cluster p values are post hoc and unadjusted. The institution hat-trace is the within-institution sum of ordinary OLS diagonal hat values. Residuals are ordinary OLS externally studentized residuals, not cluster-studentized residuals.

S1.15 Age-field version audit

The main analysis and source-tracking files were joined by the retained matching code. Age differed for 174 of 961 retained teachers (18.1%) across 90 institutions. Every nonzero main-minus-tracking difference was positive, ranging from 1 to 12 years (median 3). The migrated archive lacks evidence authenticating either version. The primary analysis retained main-file age for traceability, with no substantive interpretation of its coefficient. Omitting age changed no focal point estimate by more than .000097 (Section S1.10 and Table S20); a new complete bootstrap family was not estimated for this omission. Table S28 records the version comparison, while the true ages remain unverified.

Table S28. Age-field version comparison

Metric	Value
Matched retained records	961
Records with different age values	174
Institutions containing a difference	90
Positive main-minus-tracking differences	174
Negative main-minus-tracking differences	0
Nonzero difference: minimum / median / maximum	1 / 3 / 12 years

S1.16 Primary model specification and reporting distinctions

Observed scores and analytical levels

The primary analyses used pooled ordinary least-squares regressions with observed scores, within-between decomposition, and institution-clustered inference. The predictors comprise the institutional proxy, institution-average teacher responses, and teachers' deviations from those means, yielding level-specific estimands. The equations are observed-score regressions rather than random-intercept, random-slope, or multilevel latent-variable or structural-equation models. Section S1.4 reports separate estimator sensitivity checks.

For each focal teacher score, the between-institution component was the arithmetic mean among retained teachers in that institution; the within-institution component was the teacher's standardized score minus that mean. Standardization preceded decomposition, and both components retained that scale. The T3 AI-CTB response was the standardized observed score. Each retained teacher had unit weight in the primary OLS regressions, so larger retained clusters contributed more observations. This specification separates associations among colleagues from associations involving sampled institution means. Coefficients denoted by β are expressed in standardized observed-score variables, rather than as fully standardized multilevel latent effects.

Equation-specific predictors and covariates

Every equation included an intercept and the same control set. Continuous controls were age, teaching experience, weekly PE teaching hours, class size, and prior technology experience, each standardized across the retained cohort. Categorical controls were region, institutional stage, institutional nature, location type, gender, educational attainment, and AI training; these were represented by dummy variables with one category omitted per field. The primary model did not decompose these controls. The full Mundlak sensitivity decomposed every teacher-varying numeric and categorical control instead (Section S1.11).

The H1 equation regressed T3 AI-CTB on RDS, the within- and between-institution DSE components, and the within- and between-institution T0 AI-CTB components, together with the controls. It omitted the T2 appraisal and digital-stress indices. The additive T2 appraisal equation included RDS, both DSE components, and both T0 appraisal components. The additive T2 digital-stress equation included RDS, both DSE components, and both T0 digital-stress components. Each additive T2 equation also included the common control set.

The shared T3 outcome equation included RDS; within- and between-institution T2 appraisal and digital stress; within- and between-institution T0 appraisal and digital stress; both DSE components; both T0 AI-CTB components; and the controls. The H4a and H4b equations respectively augmented the additive T2 appraisal and stress equations with RDS × teacher-relative DSE and RDS × institution-mean DSE. All corresponding lower-order terms remained included. The teacher-relative interaction was the focal H4 estimand; the institution-mean interaction was retained as an adjustment term. These are linear conditional-association models, and baseline adjustment does not make their coefficients estimates of within-person change.

Coefficient products and their interpretation

For each H2/H3 product, a is the RDS coefficient from the corresponding additive T2 equation, and b_B is the institution-mean coefficient for that T2 response in the shared T3 equation. H2 is the appraisal product a × b_B, and H3 is the digital-stress product a × b_B. Both use a from the additive equations, separately from the H4 interaction equations. Each product summarizes the joint direction of two baseline-adjusted between-institution associations while retaining their joint sampling uncertainty. It is a descriptive estimand of the proposed temporal sequence. Interpreting it as a causal indirect effect would require identification of the exposure-response relations and assumptions about unmeasured confounding beyond temporal ordering and covariate adjustment.

H1 is estimated from its own equation without the T2 indices. Its RDS coefficient, the conditional RDS coefficient in the shared outcome equation, and the H2/H3 products are distinct estimands rather than components of a total/direct/indirect-effect decomposition; no mediated proportion is estimated. The frozen code and outputs retain identifiers such as mediator, a path, b path, and sum_indirect for computational traceability. These labels refer to the intermediate response indices, component associations, and algebraic product sum defined here.

Cluster uncertainty and multiplicity

The coefficient tables use institution-cluster sandwich standard errors with the finite-cluster CR1 correction, degrees-of-freedom correction, and Student t inference with 120 − 1 = 119 cluster degrees of freedom. The primary five-hypothesis decisions instead use the intact-institution bootstrap distribution. In each of 5,000 draws, 120 institutions were sampled with replacement and all retained teacher rows belonging to each selected institution were included. The same resample was used across every equation in the family so that dependence among the coefficient estimates was preserved. Both component coefficients were re-estimated in each draw before calculating each product.

Percentile limits are the 2.5th and 97.5th percentiles of the bootstrap distribution and are pointwise, unadjusted intervals. For a distribution of B valid estimates, the two-sided sign-proportion p value is the smaller of 1 and twice the smaller of the counts at or below zero and at or above zero, after adding one to that smaller count and dividing by B + 1. Holm adjustment was then applied to the five raw p values. Statistical support in the manuscript refers to this adjusted family, not to a component coefficient's t test or an unadjusted interval. The sign-proportion calculation is distinct from the restricted-null wild-cluster bootstrap-t check reported in Section S1.14.

Observed-score scaling and within-between decomposition were fixed before resampling. Sampling intact institutions preserves their arithmetic means and teacher deviations, but the bootstrap does not reconstruct uncertainty in the original source-summary scoring or remove measurement error in RDS and institution means.

Primary reporting and post hoc checks

The five-hypothesis family is the theory-guided primary analysis reported here. Preregistration and a timestamped plan were unavailable, so primary describes its role in the report rather than prospective prespecification. Post hoc analyses comprise baseline and estimator alternatives; influence checks and variance partitioning; level-specific correlations and stage comparisons; paired contrasts, simple slopes, and Johnson-Neyman probes; matched-position and full Mundlak analyses; source, weighting, and aggregation alternatives; and numerical and residual diagnostics. Complete alternative five-test families are identified with their own Holm-adjusted results. The remaining sensitivity intervals and p values are descriptive and unadjusted.
