# Supplementary material for: Institutional digitalization and digital stress: a four-wave observational study of teacher-reported AI-collaborative teaching in physical education
Source: Front Psychol. 2026 Oct 15;17:1973587. doi: 10.3389/fpsyg.2026.1973587 (PMC13716614; doi:10.3389/fpsyg.2026.1973587)
Supplement: Supplementary file 2 [file Data_Sheet_2.pdf]

Supplementary File S2 Revision Notes

Human-readable companion · 22 September 2026 V4

This Word/PDF companion explains the terminology addendum to the computational ZIP. The ZIP contains the executable code, numerical outputs, and original checksums.

Revision date: 22 September 2026

This addendum explains the legacy terminology in the computational-verification materials supplied with the original submission. The revised ZIP retains every original member unchanged and adds this note and a separate addendum manifest. All frozen filenames, column labels, input data, numerical estimates, provenance records, and original hash manifests are preserved.

The primary analyses use observed-score ordinary least-squares regressions with within-between decomposition and institution-clustered inference. The models distinguish institution-level and teacher-relative associations. The primary specification consists of these regressions rather than random-intercept, random-slope, or latent multilevel mediation models.

Legacy label or usage	Interpretation in the revised manuscript
mediator or intermediate-index variable names	T2 appraisal or digital-stress response indices; their position in a theoretical temporal sequence does not establish causal mediation.
a path / b path	Respectively, the RDS coefficient in an additive T2 response equation and that T2 response's institution-mean coefficient in the shared T3 AI-CTB equation. They are conditional associations.
h2 / h3	Products of the corresponding two between-institution coefficients, re-estimated together within each intact-institution bootstrap draw. These are descriptive coefficient-product estimands.
sum_indirect	The algebraic sum H2 + H3. It is not an identified total indirect effect or a proportion mediated.
mediator_adjusted_rds	The conditional RDS coefficient from the T3 equation containing both T2 indices and the full listed baseline terms. It is not an identified direct effect.

H1 is estimated from a separate T3 equation without the T2 response indices. H1, the conditional RDS coefficient, and the H2/H3 products are distinct estimands rather than components of a total/direct/indirect-effect decomposition. The RDS coefficients used in H2/H3 come from the additive T2 equations, separately from the H4 interaction equations. Original code identifiers are preserved to maintain direct links between the reported results, archived scripts, and output files.

The five-hypothesis family is the theory-guided primary family reported in the manuscript. The study was not preregistered, and no timestamped analysis plan is available. Supplementary File S1 distinguishes the primary bootstrap and Holm decisions from post hoc checks, gives all equations and controls in Section S1.16, and defines the inferential scope of each sensitivity. Age omission assesses point-estimate stability; authentication of the age versions and a new complete bootstrap family are outside its scope.

This reporting revision uses the archived numerical results and involves no new participant-level computation. Every original payload file and the original manifest retain their exact bytes. The ZIP has been repackaged to include the two revision documents, and the original ZIP checksum is recorded in REVISION_ADDENDUM_MANIFEST_20260922.json.

Current manuscript title (22 September 2026 V4): Institutional digitalization and digital stress: a four-wave observational study of teacher-reported AI-collaborative teaching in physical education.

The title now identifies a four-wave observational study. Original computational files retain their historical labels and titles; their code, numerical outputs, and checksums are unchanged. The manuscript uses observed-score OLS with within-between decomposition and institution-clustered inference.
