# Supplementary material for: Institutional digitalization and digital stress: a four-wave observational study of teacher-reported AI-collaborative teaching in physical education
Source: Front Psychol. 2026 Oct 15;17:1973587. doi: 10.3389/fpsyg.2026.1973587 (PMC13716614; doi:10.3389/fpsyg.2026.1973587)
Supplement: Supplementary file 3 [file Data_Sheet_3.pdf]

Supplementary File S3

Questionnaire wording and measurement documentation

1. Scope and scoring

This supplement documents the retained item wording, measurement schedule, and observed-score construction for the four-wave analysis. The focal institutional predictor, the reported digitalization summary (RDS), is an organizational-source proxy constructed from two retained summary fields. Its original item-level construction and administration remain unavailable in the records. The primary T3 outcome is the study-specific five-item teacher-reported AI-collaborative teaching practice score (AI-CTB). A separate 16-item teacher-perceived digitalization block contributes to the response model and descriptive convergence checks and is separate from the construction of RDS.

The Chinese statements reproduce the study-contextualized field wording in the author-provided instrument record. The English statements are translations prepared for publication and were not administered separately. Source citations identify the conceptual or item-content lineage of the adaptations, rather than equivalence to complete published scales. Independent content-validation and cross-language-equivalence procedures are undocumented. Response-model fit and reliability in Supplementary File S1 describe the study-specific response arrays; content validity, external behavioral validity, longitudinal invariance, and validation in other populations require separate evidence.

Teacher-questionnaire scores were arithmetic means of their retained items, with higher values indicating more of the named response. The current analysis used no reverse scoring. All 961 analytic records were complete on these item blocks, so partial-item prorating was unnecessary. The numeric ranges below follow the retained response documentation; no additional verbal endpoint labels are supplied.

In the JD-R-informed interpretation, RDS represents organizational context and DSE a personal resource. Functional appraisal captures teachers' evaluations, digital stress combines appraised requirements and strain, and AI-CTB describes reported work practice. Testing the canonical JD-R processes would additionally require separate measures of job resources and exposure to digital demands, which are absent from the retained data.

2. Measurement schedule and score construction

Table S3.1. Wave, timing, source, and retained item structure

Wave	Dates	Source	Retained measure	Items/fields	Numeric range
T0	7-20 Jan 2026	Teacher	Functional-appraisal baseline covariate	8 (AIA1-AIA8)	1-5
T0	7-20 Jan 2026	Teacher	Digital-stress baseline covariate	6 (DS1-DS6)	1-5
T0	7-20 Jan 2026	Teacher	AI-CTB baseline score	5 (AICTB1-AICTB5)	1-7
T1	18 Feb-3 Mar 2026	Teacher	Teacher-perceived digitalization conditions	16 (SDO1-SDO16)	1-7
T1	18 Feb-3 Mar 2026	Teacher	Digital self-efficacy (DSE)	4 (DSE1-DSE4)	1-7
T1	18 Feb-3 Mar 2026	Two organizational-source summary fields	Retained institution-head-source and IT-lead-source summaries	2 source-summary fields	1-7
T2	1-14 Apr 2026	Teacher	Functional-appraisal index	10 (AIA1-AIA10)	1-5

Wave	Dates	Source	Retained measure	Items/fields	Numeric range
T2	1-14 Apr 2026	Teacher	Digital-stress index	8 (DS1-DS8)	1-5
T3	20 May-2 Jun 2026	Teacher	Five-item teacher-report AI-CTB score	5 (AICTB1-AICTB5)	1-7

Note. Wave labels and dates refer to the retained study schedule; source-specific completion times remain unverified. The two organizational labels follow archived field titles and describe source categories. The archive lacks the information needed to verify uniform job titles, contributor identities, original organizational item wording, response processes, or independent completion. RDS was constructed solely from the two retained organizational-source summary fields.

Table S3.2. Observed-score construction and role in the current analysis

Measure	Raw-score construction	Analysis-scale construction	Higher values	Current role
Teacher-perceived digitalization conditions	Mean of 16 T1 items	Teacher score; used in the response model	Stronger perceived institutional digitalization conditions	CFA block; convergent descriptive bridge; alternative H1 operationalization
DSE	Mean of 4 T1 items	Standardized across N=961 before level decomposition	Greater digital/AI teaching self-efficacy	Teacher-level covariate; teacher-relative component used as the cross-level interaction variable
Functional appraisal	Mean of 8 T0 items; mean of 10 T2 items	Each wave-specific index standardized across N=961	More favorable functional appraisal of AI	T0 baseline covariate and T2 intermediate index
Digital stress	Mean of 6 T0 items; mean of 8 T2 items	Each wave-specific index standardized across N=961	Greater digitally related work pressure	T0 baseline covariate and T2 intermediate index
AI-CTB score	Mean of the same 5 items at T0 and T3	Each wave-specific score standardized across N=961	More teacher-reported AI involvement in teaching tasks	T0 source-matched baseline and primary T3 outcome
RDS	Equal mean of two separately standardized organizational source summaries	Composite restandardized across the 126-institution source frame	Higher reported institutional digitalization	Focal institution-level predictor

Note. The T0 and T2 appraisal and digital-stress indices overlap but differ in composition and are used for baseline adjustment rather than change scores. Supplementary File S1 reports a post hoc item-composition sensitivity restricting T2 to AIA1-AIA8 and DS1-DS6. Longitudinal measurement invariance remains untested for these indices and for the five AI-CTB items reused at T0 and T3. Teacher scores were standardized before calculation of their arithmetic institution means and individual deviations, and both components retained that scale. The means summarize sampled teachers' responses; interpreting them as shared organizational climates would require additional validation.

Table S3.3. Two-source construction of the reported digitalization summary

Component	Retained field	Transformation	Weight	Use
Institution-head-source summary	Retained 1-7 institutional source-summary field	z-standardized across 126 source institutions	0.50	RDS component
Information-technology-lead-source summary	Retained 1-7 institutional source-summary field	z-standardized across 126 source institutions	0.50	RDS component
Two-source RDS	Mean of the two source z scores	Re-standardized across 126 source institutions	-	Focal institution-level predictor

Note. For institution j, RDS_j = z_126[0.5 z_126(H_j) + 0.5 z_126(I_j)], where H_j and I_j denote the retained institution-head-source and information-technology-lead-source summaries. Standardization used the population SD. The 120 institutions with retained teacher records inherited the corresponding RDS values. This analysis-stage construction is reproducible, while the original item content and scoring behind each summary remain unavailable. Accordingly, the summaries have the status of archived proxies rather than verified infrastructure measures, established multidimensional scales, or documented means of a specific item array. Table S3.4 describes the content of the separate teacher block, which is distinct from RDS.

3. Retained teacher-questionnaire item wording

Teachers used the numeric range shown for each item block. Retained item codes provide a direct link to the analysis files. Historical labels such as SDO and AIA identify item positions, while the manuscript uses the corresponding study-specific construct names. Content-domain labels summarize item content; they were not used to define, score, or validate subscales or separate latent dimensions.

Table S3.4. Teacher-perceived digitalization conditions (16 items; numeric range 1-7)

Code	Content domain	English publication wording	Chinese field wording	Wave/reporter
SDO1	Digital technology scope	My school uses digital technologies to expand the provision of curriculum and teaching services.	本校利用数字技术扩大课程与教学服务的供给。	T1 teacher
SDO2	Digital technology scope	My school uses digital technologies to add new functions to existing teaching or administrative services.	本校利用数字技术为既有教学或管理服务增加新功能。	T1 teacher
SDO3	Digital technology scope	My school treats digital technologies as a key resource for expanding teaching and learning support.	本校把数字技术视为拓展教学与学习支持的关键资源。	T1 teacher
SDO4	Digital technology scope	My school uses digital technologies to create additional value for teachers and students.	本校利用数字技术为教师和学生创造额外价值。	T1 teacher
SDO5	Digital capabilities	Teachers and relevant staff in my school understand the potential uses and consequences of digital technologies.	本校教师及相关人员了解数字技术的潜在用途及其影响。	T1 teacher

Code	Content domain	English publication wording	Chinese field wording	Wave/reporter
SDO6	Digital capabilities	My school continuously improves teachers' relevant capabilities around digitalization goals.	本校围绕数字化目标持续提升教师的相关能力。	T1 teacher
SDO7	Digital capabilities	Teachers and relevant staff in my school can overcome challenges arising from the use of digital technologies.	本校教师及相关人员能够克服使用数字技术时出现的困难。	T1 teacher
SDO8	Digital capabilities	Teachers and relevant staff in my school can use the main functions of the digital technologies available to perform their work.	本校教师及相关人员能够运用现有数字技术的主要功能完成工作。	T1 teacher
SDO9	Digital ecosystem coordination	My school collaborates with external technology service providers to advance digital construction.	本校与外部技术服务提供方协作推进数字化建设。	T1 teacher
SDO10	Digital ecosystem coordination	My school integrates external partners with different responsibilities into its digitalization work.	本校把职责不同的外部伙伴整合进数字化工作。	T1 teacher
SDO11	Digital ecosystem coordination	My school attracts partners that create educational value for the school and its wider digital ecosystem.	本校能够吸引为学校及其数字生态创造教育价值的合作伙伴。	T1 teacher
SDO12	Digital ecosystem coordination	My school creates mechanisms that encourage digital partners to share expertise and resources.	本校建立机制，促进数字合作伙伴共享专业能力与资源。	T1 teacher
SDO13	Digital architecture configuration	In response to digital change, my school continuously adapts relevant organizational arrangements.	为应对数字化变化，本校持续调整相关组织安排。	T1 teacher
SDO14	Digital architecture configuration	My school's teaching and administrative processes can adapt to digital change.	本校的教学与管理流程能够适应数字化变化。	T1 teacher
SDO15	Digital architecture configuration	My school uses digital technologies to digitize internal teaching and administrative processes.	本校利用数字技术推进内部教学与管理流程数字化。	T1 teacher
SDO16	Digital architecture configuration	My school's information infrastructure and systems support a wide range of educational uses and internal collaboration.	本校的信息基础设施和系统能够支持多种教育用途及内部协作。	T1 teacher

Note. The historical SDO item codes refer to this teacher questionnaire block, not to the two-source RDS predictor.

Table S3.5. Digital self-efficacy (4 items; numeric range 1-7)

Code	Content domain	English publication wording	Chinese field wording	Wave/reporter
DSE1	Instructional design	I am confident that I can design PE classroom activities that include AI support.	我有信心设计包含 AI 支持的体育课堂活动。	T1 teacher
DSE2	Learning monitoring	I am confident that I can use digital tools or AI systems to monitor students' progress in PE learning.	我有信心使用数字工具或 AI 系统监测学生的体育学习进展。	T1 teacher
DSE3	Classroom diagnosis	I am confident that I can use digital tools or AI systems to diagnose students' movement-learning difficulties during PE lessons.	我有信心使用数字工具或 AI 系统诊断学生在体育课中的动作学习困难。	T1 teacher
DSE4	Assessment	I am confident that I can use digital systems to assess students' motor performance and classroom participation.	我有信心使用数字系统评价学生的动作表现和课堂参与。	T1 teacher

Table S3.6. Study-specific functional-appraisal index (10-item T2 bank; numeric range 1-5)

Code	Content domain	English publication wording	Chinese field wording	Wave
AIA1	Perceived adaptability	AI can learn from experience in PE teaching.	AI 能够从体育教学经验中学习。	T0 and T2
AIA2	Perceived adaptability	AI can improve its analyses and support through continued use in PE teaching.	AI 能够在持续用于体育教学的过程中改进其分析与支持。	T0 and T2
AIA3	Perceived adaptability	AI can quickly adjust its analytic approach in response to changes in PE teaching tasks.	AI 能够根据体育教学任务的变化快速调整其分析方式。	T0 and T2
AIA4	Perceived quality	AI can provide a sufficiently complete set of information for PE teaching decisions.	AI 能够为体育教学决策提供较为完整的信息。	T0 and T2
AIA5	Perceived quality	The information produced by AI for PE teaching is generally accurate.	AI 为体育教学生成的信息总体上是准确的。	T0 and T2
AIA6	Perceived quality	The instructional feedback and data-based judgments provided by AI are reliable.	AI 提供的教学反馈和基于数据的判断是可靠的。	T0 and T2
AIA7	Perceived quality	The information provided by AI for PE teaching is sufficiently up to date.	AI 为体育教学提供的信息能够保持较新的状态。	T0 and T2

Code	Content domain	English publication wording	Chinese field wording	Wave
AIA8	Personal utility	Using AI in PE teaching increases my confidence in solving complex instructional problems.	在体育教学中使用 AI 增强了我解决复杂教学问题的信心。	T0 and T2
AIA9	Personal utility	Using AI in PE teaching gives me a worthwhile sense of accomplishment.	在体育教学中使用 AI 会给我带来有价值的成就感。	T2 only
AIA10	Personal utility	Using AI gives me greater control over my PE teaching work.	使用 AI 使我对体育教学工作拥有更强的掌控感。	T2 only

Note. AIA1-AIA8 formed the T0 baseline covariate, and AIA1-AIA10 formed the T2 index. The selected content covers perceived adaptability, perceived quality, and personal utility, including confidence, accomplishment, and control. It captures functional evaluations of AI. Available job resources, realized autonomy, and occupational engagement are distinct constructs, and the content labels used here denote descriptive domains rather than separately validated subscales.

Table S3.7. Study-specific digital-stress index (8-item T2 bank; numeric range 1-5)

Code	Content domain	English publication wording	Chinese field wording	Wave
DS1	Workload/time pressure	Calibrating AI devices or wearable systems on an outdoor playground makes me feel pressed for time.	在室外场地校准 AI 设备或可穿戴系统会使我感到时间紧迫。	T0 and T2
DS2	Workload/time pressure	Preparing and troubleshooting digital or AI equipment adds substantially to my PE teaching workload.	准备并排查数字化或 AI 设备问题明显增加了我的体育教学工作量。	T0 and T2
DS3	Vigilance/connection burden	Continuously checking platform messages, device connections, and data uploads before a PE class makes me feel additionally burdened.	体育课前持续检查平台消息、设备连接和数据上传会给我带来额外负担。	T0 and T2
DS4	Vigilance/availability	I feel uneasy if I fail to check digital prompts or system feedback related to the class in a timely manner.	如果未能及时查看与课堂有关的数字提示或系统反馈，我会感到不安。	T0 and T2
DS5	Platform pressure	I feel pressured to respond promptly to school messages or tasks delivered through digital platforms.	我会因需要及时回应学校数字平台中的消息或任务而感到压力。	T0 and T2
DS6	Platform pressure	Platform reporting, uploading, or data-entry requirements create additional pressure in my work.	平台填报、上传或数据录入要求给我的工作带来额外压力。	T0 and T2

Code	Content domain	English publication wording	Chinese field wording	Wave
DS7	Digital complexity	Learning to use increasingly complex AI or digital teaching tools requires too much effort from me.	学习使用日益复杂的 AI 或数字教学工具需要我投入过多精力。	T2 only
DS8	Complexity/uncertainty	Frequent system updates or technical problems disrupt my PE teaching work.	频繁的系统更新或技术问题会干扰我的体育教学工作。	T2 only

Note. DS1-DS6 formed the T0 baseline covariate, and DS1-DS8 formed the T2 index. DS1 and DS3-DS6 concern pressure, burden, or unease; DS2 concerns added workload, DS7 excessive effort, and DS8 disruption. The bank combines stress-response wording with appraised work requirements. Digital stress denotes this study-specific index of perceived digital work pressure. Conceptually, digital job demands concern work requirements and strain concerns psychological responses; independent measures would be needed to separate them. Technostress refers to the wider research literature, while the present bank remains a study-specific adaptation.

Table S3.8. Five-item study-specific teacher-report AI-CTB score (numeric range 1-7)

Code	Content domain	English publication wording	Chinese field wording	Wave
AICTB1	Decision making	AI participates in my decision-making process in PE teaching.	AI 参与了我在体育教学中的决策过程。	T0 and T3
AICTB2	Prediction	AI participates in my prediction of students' motor performance and instructional effectiveness.	AI 参与了我对学生动作表现和教学效果的预测。	T0 and T3
AICTB3	Problem solving/diagnosis	AI participates in my process of solving or diagnosing problems in PE teaching.	AI 参与了我解决或诊断体育教学问题的过程。	T0 and T3
AICTB4	Information identification/evaluation	AI participates in my identification and evaluation of information relevant to PE teaching.	AI 参与了我识别和评价体育教学相关信息的过程。	T0 and T3
AICTB5	Risk/opportunity recognition	AI participates in my identification of classroom risks, movement deviations, or areas for training improvement.	AI 参与了我识别课堂风险、动作偏差或训练改进空间的过程。	T0 and T3

Note. The T3 mean of these five items was the primary outcome, with the same five-item T0 score providing baseline adjustment. The tasks concern teachers' professional decisions, predictions, diagnosis, information evaluation, and recognition of risks or improvement opportunities. The score therefore situates self-reported AI use in work practice beyond adoption intention. Its interpretation is confined to these task reports: independent behavior, task frequency with verified verbal anchors, collaboration quality, teaching effectiveness, employee performance or well-being, and student outcomes were not measured. Higher scores indicate more reported AI involvement, without implying better teaching or more effective collaboration.

4. Measurement lineage and contextual adaptation

The adaptations translate organizational and employee task referents into institutional settings and PE teachers' instructional decisions, movement diagnosis, assessment, and risk recognition. These work referents can be inspected in the retained Chinese wording. The teacher digitalization block concerns educational provision and institutional arrangements. DSE addresses confidence in concrete teaching tasks, including a diagnosis item developed for this study. Functional appraisal covers AI adaptability, quality, and personal utility, while the digital-stress bank addresses burden, workload, effort, and disruption in digitally supported PE teaching. AI-CTB retains five task categories from employee-AI collaboration research (Kong et al., 2023), adapted to teaching. Table S3.9 documents the selected and reworded content and the status of each adaptation. Independent expert content-validity assessment, cognitive testing, and cross-language validation remain undocumented in the archive.

Table S3.9. Measurement lineage and status of the study adaptations

Study-specific block	Measurement lineage	Status of the present wording
Teacher-perceived digitalization conditions	Selected content spanning the digital-orientation domains described by Kindermann et al. (2024)	Contextually adapted 16-item teacher block; not the original 19-item instrument and not a component of RDS
DSE	Three task themes from teachers' ICT self-efficacy for instructional purposes - lesson/activity design, learning-progress monitoring, and assessment (Hatlevik and Hatlevik, 2018) - with broader conceptual grounding in computer self-efficacy (Compeau and Higgins, 1995) and teacher digital competence (Tondeur et al., 2023)	Three task themes were contextually adapted for digital and AI-supported PE teaching; the classroom-diagnosis item was developed for this study. The four-item set is not the Compeau scale, the HeDiCom framework, or a validated short form.
Functional appraisal	Perceived adaptability, perceived quality, and personal-utility content from the six-dimensional Attitudes Towards Artificial Intelligence at Work scale (Park et al., 2024)	Selected and contextually adapted 10-position item bank; not the complete source instrument or a validated short form
Digital stress	Multidimensional digital-stress and occupational-technostress research (Hall et al., 2021; Funke et al., 2026; Ragu-Nathan et al., 2008)	Study-contextualized PE teaching item bank informed by multiple sources; not a shortened version of any single cited scale
Teacher-report AI-CTB	Five-item employee-AI collaboration measure developed by Kong et al. (2023)	Five item contents/task categories contextually adapted and re-referenced to PE teaching tasks; not an independently observed behavioral measure and not five validated subdimensions.

5. References

Compeau, D. R., and Higgins, C. A. (1995). Computer self-efficacy: development of a measure and initial test. MIS Quarterly 19, 189-211. doi: 10.2307/249688

Funke, C., Rothert-Schnell, C., Walsh, G., Mangiò, F., Pedeliento, G., and Takahashi, I. (2026). The digital stress scale: cross-cultural application, validation, and development of a short scale. Review of Managerial Science 20, 1229-1265. doi: 10.1007/s11846-025-00906-w

Hall, J. A., Steele, R. G., Christofferson, J. L., and Mihailova, T. (2021). Development and initial evaluation of a multidimensional digital stress scale. Psychological Assessment 33, 230-242. doi: 10.1037/pas0000979

Hatlevik, I. K. R., and Hatlevik, O. E. (2018). Examining the relationship between teachers' ICT self-efficacy for educational purposes, collegial collaboration, lack of facilitation and the use of ICT in teaching practice. Frontiers in Psychology 9:935. doi: 10.3389/fpsyg.2018.00935

Kindermann, B., Schmidt, C. V. H., Fengel, F., and Strese, S. (2024). Expanding the boundaries of digital orientation research: scale development and validation. Journal of Business Research 185:114895. doi: 10.1016/j.jbusres.2024.114895

Kong, H., Yin, Z., Baruch, Y., and Yuan, Y. (2023). The impact of trust in AI on career sustainability: the role of employee-AI collaboration and protean career orientation. Journal of Vocational Behavior 146:103928. doi: 10.1016/j.jvb.2023.103928

Park, J., Woo, S. E., and Kim, J. (2024). Attitudes towards artificial intelligence at work: scale development and validation. Journal of Occupational and Organizational Psychology 97, 920-951. doi: 10.1111/joop.12502

Ragu-Nathan, T. S., Tarafdar, M., Ragu-Nathan, B. S., and Tu, Q. (2008). The consequences of technostress for end users in organizations: conceptual development and empirical validation. Information Systems Research 19, 417-433. doi: 10.1287/isre.1070.0165

Tondeur, J., Howard, S., Van Zanten, M., Gorissen, P., Van der Neut, I., Uerz, D., et al. (2023). The HeDiCom framework: higher education teachers' digital competencies for the future. Educational Technology Research and Development 71, 33-53. doi: 10.1007/s11423-023-10193-5
